# Dynamics of the Phosphoinositide 3-Kinase p110δ Interaction with p85α and Membranes Reveals Aspects of Regulation Distinct from p110α

**DOI:** 10.1016/j.str.2011.06.003

**Published:** 2011-08-10

**Authors:** John E. Burke, Oscar Vadas, Alex Berndt, Tara Finegan, Olga Perisic, Roger L. Williams

**Affiliations:** 1 Medical Research Council, Laboratory of Molecular Biology, Cambridge CB2 0QH, UK

## Abstract

Phosphoinositide 3-kinase δ is upregulated in lymphocytic leukemias. Because the p85-regulatory subunit binds to any class IA subunit, it was assumed there is a single universal p85-mediated regulatory mechanism; however, we find isozyme-specific inhibition by p85α. Using deuterium exchange mass spectrometry (DXMS), we mapped regulatory interactions of p110δ with p85α. Both nSH2 and cSH2 domains of p85α contribute to full inhibition of p110δ, the nSH2 by contacting the helical domain and the cSH2 via the C terminus of p110δ. The cSH2 inhibits p110β and p110δ, but not p110α, implying that p110α is uniquely poised for oncogenic mutations. Binding RTK phosphopeptides disengages the SH2 domains, resulting in exposure of the catalytic subunit. We find that phosphopeptides greatly increase the affinity of the heterodimer for PIP2-containing membranes measured by FRET. DXMS identified regions decreasing exposure at membranes and also regions gaining exposure, indicating loosening of interactions within the heterodimer at membranes.

## Highlights

► Both nSH2 and cSH2 of p85α inhibit p110δ, and pY peptides or mutations activate ► DXMS mapped dynamic changes in free p110δ and p110δ/p85 with and without pY peptides ► pY peptide increases p110δ/p85 affinity for lipids; interactions were mapped by DXMS ► cSH2 point mutation in p85α activates p110δ and p110β, but not p110α

## Introduction

Phosphoinositide 3-kinases (PI3Ks) are a family of enzymes that catalyze the phosphorylation of the D3-hydroxyl of inositol phospholipids. The lipid products of the reaction act as second messengers and lead to downstream recruitment of phosphoinositide binding proteins, which orchestrate many important functions including cell proliferation and survival (Vanhaesebroeck et al., 2010). PI3Kδ is upregulated in some leukemias, and a specific inhibitor of it (CAL-101) has entered clinical trials for chronic lymphocytic leukemia (Lannutti et al., 2010). This isoform is abundant in leukocytes and has important roles in immunity, inflammation and allergy. Like other class IA PI3Ks, it is an obligate heterodimer of a p110 catalytic subunit (p110δ) and a p85-type regulatory subunit (p85α, p55α, p50α, p85β, or p55γ) and produces PtdIns(3,4,5)P_3_ lipids in vivo (Cantley, 2002; Geering et al., 2007). The p110α catalytic subunit is one of the most frequently mutated oncogenes in human tumors, and PI3K inhibition is a major pharmacological target for drug discovery (Samuels et al., 2004; Wong et al., 2010). The cancer-associated p110α mutations upregulate the enzyme activity by different mechanisms (Huang et al., 2008; Mandelker et al., 2009; Miled et al., 2007; Wu et al., 2009; Zhao and Vogt, 2008). Because any one of the regulatory subunits can interact tightly with any of the class IA catalytic subunits, it is generally assumed that all p110 subunits are inhibited in the same way. The importance of understanding isoform-specific regulation of PI3Ks by p85 led us to examine this assumption.

All of the p110 subunits consist of an adaptor-binding domain (ABD), a Ras binding domain (RBD), a C2 domain, a helical domain, and a kinase domain split into an N-lobe and a C-lobe (Berndt et al., 2010; Huang et al., 2007; Walker et al., 1999). The p85 subunits consist of an N-terminal SH3 domain, a breakpoint cluster region (BCR) homology domain flanked by two proline-rich regions, and two SH2 domains, nSH2 and cSH2, separated by a coiled-coil region termed the inter-SH2 domain (iSH2) (see Figure S1 available online). The ABD domain has been shown to be necessary and sufficient to bind the regulatory subunit, through interactions with the iSH2 domain (Dhand et al., 1994). The regulatory subunits stabilize the catalytic subunit, inhibit its basal activity, and activate PI3K in the presence of phosphorylated tyrosine (pY) sequences in activated receptor tyrosine kinases (RTKs) and adaptor proteins (Yu et al., 1998a, 1998b). The N-terminal SH2 domain (nSH2) in combination with the iSH2 domain is the minimal construct of p85α required to inhibit the activity of p110α (Yu et al., 1998a). This inhibition can be relieved by RTK pY peptides. Constructs of the p110α isoform with oncogenic mutations in the helical domain display increased activity compared to the wild-type enzyme and have no further increase in activity on RTK pY peptide binding (Carson et al., 2008; Chaussade et al., 2009). However, regulation of the p110δ isoform by p85 and RTK pY peptides has not been examined.

Understanding the dynamics between the catalytic and regulatory subunits is central to understanding PI3K activity. The structures of both the isolated nSH2 and cSH2 domains of p85α with and without RTK pY peptides are known (Hoedemaeker et al., 1999; Nolte et al., 1996), as well as the structure of the niSH2 fragment bound to the full-length p110α (Mandelker et al., 2009). The nSH2 domain binds the helical domain through the surface required for pY peptide binding, and consequently the presence of a pY peptide breaks the inhibitory contacts between the catalytic and regulatory subunits (Mandelker et al., 2009; Miled et al., 2007). Many of the cancer linked mutations identified are located at two separate interfaces between the p110 catalytic and p85α subunits, one between the iSH2 domain of p85 and the C2 domain of p110α, and one between the nSH2 domain of p85 and the helical domain of p110α (Huang et al., 2007; Miled et al., 2007; Wu et al., 2009). However, no structural information exists for possible interactions between the nSH2 and cSH2 domains with p110δ.

The recently published structures of the free p110δ catalytic subunit (Berndt et al., 2010) contributed to developing isotype-specific inhibitors, but they shed no light on how this isoform is regulated by p85. Toward this end, we used deuterium exchange mass spectrometry (DXMS) to explore the interactions between the p110δ and p85α. Due to recent advances in instrumentation and analysis, DXMS has become a burgeoning and powerful method for examining protein folding, protein-protein contacts, protein-ligand binding, and conformational changes (Engen, 2009; Zhang et al., 2010). This technique has also been used to study membrane associated enzymes interacting with phospholipid vesicles (Burke et al., 2008a, 2008b; Hsu et al., 2009).

Using DXMS, we have localized p110δ regions interacting with the iSH2, nSH2 and cSH2 domains. Our lipid kinase assays suggest that full inactivation of p110δ requires a p85 construct encompassing both SH2 domains (nicSH2). Binding of RTK pY peptides to the SH2 domains exposed p110δ regions protected by the nSH2 and cSH2 domains to deuterium exchange, leading to full activation of all constructs. DXMS results enabled us to create a p85 point mutant that exposes the proposed cSH2 binding site on p110δ, circumventing downregulation by the cSH2 in vitro and increasing downstream signaling in cells. Importantly, this same mutation also activates p110β, but not p110α. Combining this cSH2 mutation with an oncogenic mutation in the nSH2, which exposed the helical domain, results in a p85 that fully activated p110δ. Using FRET measurements, we find that p110δ/p85α complex binds very poorly to lipid vesicles mimicking plasma membrane composition, but pY peptides greatly increased affinity of the complex for lipids, especially when the vesicles contained PIP2 substrate. DXMS also enabled us to identify for the first time specific regions in both the catalytic and regulatory subunits that interact with lipid membranes. Interestingly, some regions of the ABD domain and iSH2 become more exposed in the presence of membrane, indicating that loosening of interactions within the complex accompanies membrane binding.

## Results

### The cSH2 of p85α Plays an Important Regulatory Role for p110δ

**Figure 1:**
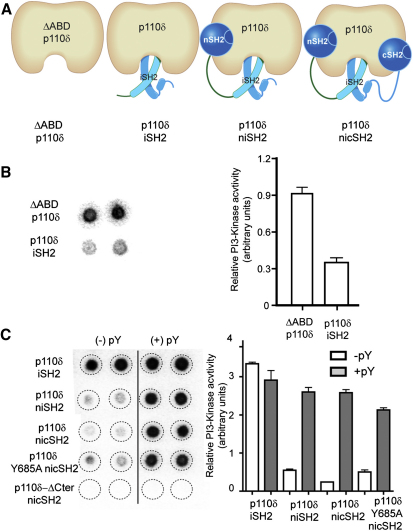
Kinase Activity of p110δ in the Presence of p85α Constructs and PDGFR pY (A) p110δ/p85α constructs tested for lipid kinase activity. (B) Kinase activity of ΔABD-p110δ compared with p110δ /iSH2 p85α. Assays measured ^32^P-PIP3 production in the presence of 0.5 nM enzyme, 100 μM ATP, and 5% PIP2 lipid vesicles at a concentration of 1 mg/ml. The left panel illustrates the autoradiogram of the filter (duplicate spots) and the right shows the quantitation of the spots. Kinase assays were performed in duplicate and repeated twice. The error bars show the standard deviation (SD). (C) In vitro kinase assay results for various p110δ and p85α constructs are shown. Assays measured ^32^P-PIP3 production in the presence of 5 nM enzyme, 100 μM ATP, and 5% PIP2 vesicles at a concentration of 1 mg/ml, +/− 10 μM PDGFR pY. Kinase assays were performed in duplicate and repeated twice.

The regulation of p110α by p85 has been thoroughly studied (Yu et al., 1998a, 1998b), and it has been assumed that the same mechanism of regulation would govern all class IA enzymes, including p110δ. To examine this assumption, we first performed lipid kinase assays to test which of the domains of p85α contribute to inhibition of the basal activity of p110δ. For the assays we used vesicles containing 5% PIP2 and a defined mixture of lipids mimicking natural membranes (20% PS/45% PE/15% PC/10% cholesterol and 5% sphingomyelin) similar in composition to previous studies examining plasma membrane interactions (Corbin et al., 2007; Maier et al., 1999). We used several truncated variants of the p85α regulatory subunit: iSH2 (residues 434–600), which has only the iSH2 domain; niSH2 (residues 322–600), which has both the nSH2 and iSH2 domains; and nicSH2 (residues 322–724), which has the nSH2, iSH2, and cSH2 domains (Figure 1A; Figure S1A). The regulatory subunits were coexpressed and copurified with the full length p110δ catalytic subunit. To express the catalytic subunit free from a regulatory subunit we used the ΔABD-p110δ construct (ΔABD-p110δ, residues 106–1044), because the full-length p110δ in the absence of a regulatory subunit is insoluble (Berndt et al., 2010). ΔABD-p110δ had a ∼2.5-fold higher activity compared to p110δ+iSH2 (Figure 1B). Because both the ABD domain and iSH2 domain are in contact with the kinase domain, either could be inhibiting PI3K activity in the p110δ+iSH2 complex. The p110δ+niSH2 complex was inhibited only ∼5-fold compared to p110δ+iSH2 (Figure 1C). However, the p110δ+nicSH2 complex was inhibited ∼15-fold compared to p110δ+iSH2, making it significantly less active than the p110δ+niSH2 complex. The inhibition of p110δ by the cSH2 domain, in the context of the nicSH2 construct, stands in sharp contrast with previous studies on p110α, which showed that the minimal construct necessary for full inhibition was the niSH2, and that the cSH2 does not inhibit the basal activity of p110α, even though it was shown to be necessary for full activation by a pY peptide (from IRS-1) in the context of full length p85 (Rordorf-Nikolic et al., 1995; Yu et al., 1998a).

We also tested if the presence of PDGFR bis-phosphorylated pY peptide (residues 735–767, with pY740 and pY751, referred to afterwards as PDGFR pY) can relieve inhibition imposed by both the nSH2 and cSH2 domains. Indeed, we found that the presence of PDGFR pY does fully relieve inhibition from both the nSH2 and cSH2, which brings the enzyme activity for all iSH2-containing complexes to a similar activated level, close to the activity of the p110δ+iSH2 (Figure 1C). The highest activation occurred with the nicSH2, which was activated ∼13-fold by the PDGFR pY. The fold of activation is critically dependent on the nature of the lipid substrate. However, the activation we see is in line with studies on purified full length p110δ/p85α complex on PIP2-containing lipid vesicles showing a 20–40-fold activation on addition of a pY peptide (Maier et al., 1999). We carried out DXMS experiments to detect changes in protein dynamics that might explain inhibition by both the nSH2 and cSH2 domains of the regulatory subunit, and activation by PDGFR pY binding.

### Digestion of p110δ and p85α Constructs/Global Deuterium Exchange of p110δ and p85α

The optimized peptide digestion map of the p110δ (Figure S1B) was composed of 116 individual peptic peptides, covering ∼83% of the sequence of the catalytic subunit, whereas the map of the p85α nicSH2 regulatory subunit consisted of 53 peptides, covering ∼85% of the sequence of the regulatory subunit. Four different enzyme complexes were studied by deuterium exchange: p110δ+p85α nicSH2 in the presence and absence of PDGFR pY, p110δ+p85α iSH2, and p110δ alone (ΔABD-p110δ). No experiments were performed on the ΔABD-p110δ in the presence of p85α constructs due to previous work showing no detectable binding of p85 by p110 without the ABD domain (Dhand et al., 1994). All heterodimers had an equal ratio of p110 and p85. The global exchange profile of ΔABD-p110δ is shown modeled onto the recently solved crystal structure of the p110δ catalytic subunit (Berndt et al., 2010) (Figure S2). The global exchange profile of the p85α nicSH2 was modeled onto the structures of the isolated nSH2 (residues 323–432) and isolated cSH2 (residues 613–720) domains in complexes with PDGFR-pY, and onto the isolated iSH2 (residues 431–600) (Hoedemaeker et al., 1999; Miled et al., 2007; Nolte et al., 1996) (Figure S2).

### Differences in Exchange of p85α in the Presence of PDGFR pY

**Figure 2:**
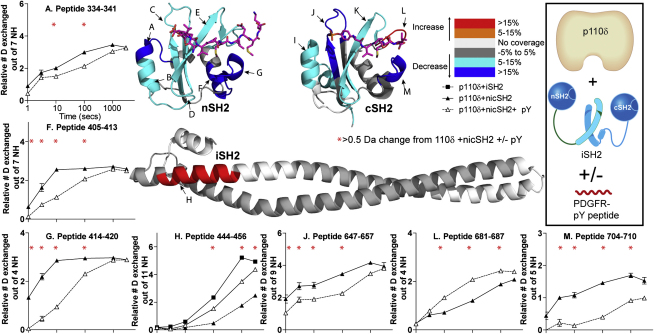
Changes in Deuteration Levels of the p85α nicSH2 Construct (Bound to p110δ) in the Presence of 40 μM PDGFR pY Peptides spanning p85α (A–M) that showed >0.5 Da changes in deuteration level in the presence and absence of PDGFR pY for the p110δ+nicSH2 complex are mapped onto the structures (2VIY for the iSH2, 2IUI for the nSH2, and 1H9O for the cSH2). The percent change mapped on the structure according to the legend is the highest deuterium exchange difference change seen at any time point in the analysis. The structures of PDGFR pY bound to the nSH2 and cSH2 are colored purple. Only peptides that showed >10% change for more than two time points are graphed and shown below the structures. Experiments were performed in duplicate, and graphs are shown ± SD. All other peptides with changes >0.5 Da are shown in Figure S3. The graphs are labeled (^∗^) for any time point with a >0.5 Da change for the p110δ+nicSH2 complex +/− PDGFR pY (see also Figure S3).

Differences in exchange rates for the p85α nicSH2 (bound to p110δ) in the presence and absence of a PDGFR pY were mapped onto the crystal structures of the nSH2, iSH2, and cSH2 of p85α (Figure 2; Figure S3). It is important to note that many of the deuterium exchange differences caused by p85α constructs and PDGFR pY binding mapped on the crystal structures are on the order of 1.0 Da within peptides that can incorporate >10 deuterons. Without sufficient overlapping peptides it is impossible to precisely locate the position of these changes. Although peptides with changes are colored in all figures, it is possible that only one to two amides in these regions are affected. The nSH2 and cSH2 domains showed nearly global decreases in exchange in the presence of PDGFR pY. This agrees with previous DXMS work that shows global decreases in exchange for isolated SH2 domains bound to pY peptides (Engen et al., 1999). We were most interested in looking for peptides that have increases in exchange in the presence of PDGFR pY, as these might indicate interfacial regions between p110δ and p85α. Two p85α peptides, 444–456 in the beginning of the iSH2 and 681–687 in the cSH2, showed increases in exchange in the presence of PDGFR pY. Peptide 444–456 also showed an increase in exchange in the p110δ+iSH2 complex in comparison with the p110δ+nicSH2. An increase in exchange was also seen with peptides spanning the C-terminal end of the iSH2 (546–581) in the absence of the nSH2 and cSH2 domains, suggesting that the coiled-coil becomes more protected from exchange in the presence of these two SH2 domains (Table S1B).

Peptide 681–687 in the cSH2 also showed an increase in exchange (1.0 Da) in the presence of PDGFR pY. We hypothesized that this region in the cSH2 might form a contact surface for p110δ, and that this interaction is broken in the presence of PDGFR pY. No region on the nSH2 had an increase in exchange in the presence of PDGFR pY. We would like to emphasize that no change in exchange would be expected if the PDGFR pY protects the same regions as are protected by p110δ in the absence of PDGFR pY. Previous crystallographic work on p110α would predict that this is exactly the case for the nSH2 (Mandelker et al., 2009). We find that the cSH2 showed an increase in exchange on PDGFR pY binding (region 681–687), suggesting that the PDGFR pY binding surface of the cSH2 and the inhibitory contact surface with p110 do not completely coincide, in contrast to the nSH2, where they do.

### Differences in Exchange of p110δ in the Presence of p85α and PDGFR pY

**Figure 3:**
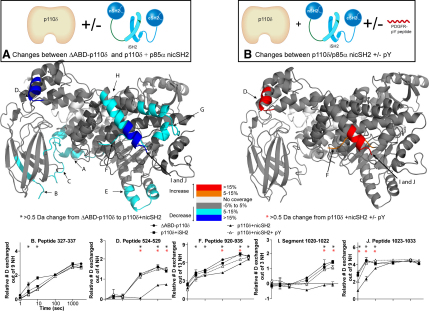
Changes in Deuteration Levels of p110δ Catalytic Subunit in the Presence of Both p85α and PDGFR pY (A) Peptides spanning p110δ (labeled A–I) that showed >0.5 Da changes in deuteration level in the presence and absence of the p85α nicSH2 are mapped onto the ΔABD-p110δ structure (2WXH) according to the legend. Peptides that showed >10% change for more than two time points are graphed and shown below the figure. Experiments were performed in duplicate, and graphs are shown ± SD. All other peptides with changes >0.5 Da are shown in Figure S4. (B) Peptides spanning the p110δ catalytic subunit in the p110δ+nicSH2 complex that showed >0.5 Da changes in deuteration level in the presence and absence of 40 μM PDGFR pY are mapped onto the structure. The percent change mapped on the structure according to the legend is the highest deuterium exchange difference change seen at any time point in the analysis. The area from 1020–1022 is named a segment to denote that this data was generated by subtraction of the deuterium level of peptide 1001–1019 from peptide 1001–1022. The graphs are labeled (^∗^) for any time points with a >0.5 Da change between the ΔABD-p110δ and p110δ+nicSH2 constructs and (^∗^) for any time point with a >0.5 Da change for the p110δ+nicSH2 +/− PDGFR pY (see also Figure S4).

To gain an insight into regions on p110δ that are in contact with the p85 subunit, we determined differences in exchange rates for p110δ in the presence of p85α constructs. Distinct regions in p110δ showed differences in exchange between the free catalytic subunit (ΔABD-p110δ) and p110δ bound to the nicSH2 of p85α (Figure 3A; Figure S4). By comparing the exchange differences between the p110δ+iSH2 complex and the p110δ+nicSH2 complex we were able to localize changes caused by binding to the iSH2 domain, and changes that were caused by the nSH2 and cSH2 domains.

#### Differences in Exchange Caused by the iSH2

Four regions of p110δ showed decreases in exchange between the ΔABD-p110δ and the p110δ+iSH2 complex: 120–138 (in the linker between the ABD and RBD), 327–337 and 453–468 (C2 domain), and 698–713 (in the N-lobe of the kinase domain) (Figure 3A; Figure S4). The region 120–138 is located near the interface with the ABD domain, and the change in deuteration is most likely the consequence of the ABD deletion. Peptides 327–337, 453–468, and 698–713 of p110δ are all located at or near the crevice where the iSH2 domain binds in the p110α/iSH2 structure (Huang et al., 2007). There was also a slight decrease in exchange in peptide 920–935 spanning the substrate-binding loop.

#### Helical Domain Changes Caused by nicSH2 and PDGFR pY

Several distinct peptides showed decreases in exchange in the presence of the p110δ+nicSH2 complex compared to the p110δ+iSH2 complex. One of these peptides, 524–529, is located in the helical domain and showed a decrease in exchange (>1.0 Da) (Figure 3A). This peptide contains E525, equivalent to E545 in p110α, which has been shown biochemically (Miled et al., 2007) and structurally (Mandelker et al., 2009) to be important in binding the nSH2. Experiments were also carried out with the p110δ+nicSH2 complex in the presence of PDGFR pY (Figure 3B). The presence of PDGFR pY exposed this region to exchange to the same extent as the free catalytic domain, suggesting that PDGFR pY binding breaks the contacts between the nSH2 and p110δ. This is consistent with our lipid kinase assays showing that the presence of PDGFR pY activates the p110δ+niSH2 complex to the same level as the p110δ+iSH2 complex, and agrees with previous results for p110α (Miled et al., 2007).

#### Kinase Domain Changes Caused by nicSH2 and PDGFR pY

Peptides spanning the substrate-binding loop (920–935) and the C-terminal end of the kinase domain (1010–1019, 1020–1022, and 1023–1033) also showed decreases in exchange in the presence of the p110δ+nicSH2 complex compared to the p110δ+iSH2 complex (Figure 3A). None of these peptides are near the nSH2 contact site with the catalytic domain of p110α, and we postulate that they may form a contact site for the cSH2 in p110δ. The largest decrease in exchange (2.0 Da) was in the peptide from 1023–1033, but only at early time points, with no changes in exchange after 100 s of on-exchange. These changes were equally distributed through this region, because peptides 1023-1027 and 1028-1033 showed decreases (>0.7 Da) in exchange (Figure S4). This region spans the end of helix kα11 and six residues following this helix, which are disordered in the structure of p110δ. This area forms the “elbow” in p110γ and class III Vps34 isoforms that connects the two C-terminal helices kα11 and kα12 (Miller et al., 2010; Walker et al., 1999). It has been recently shown that deletion of the kα12 in Vps34 abolishes the lipid kinase activity (Miller et al., 2010), so we tested an analogous deletion for p110δ/p85α nicSH2 (Δ1028-1044 in p110δ). This deletion completely abolishes p110δ lipid kinase activity (Figure 1C). Therefore, the DXMS experiments show that this region critical for catalysis has one of the largest decreases in exposure caused by nicSH2. Peptide 920–935 that spans the distal half of the substrate-binding loop (residues 911–36 in p110δ) showed a decrease in exchange (>1.5 Da). The presence of PDGFR pY exposed this peptide as well as the C-terminal peptides 1020–1022 and 1023–1033 in p110δ+nicSH2 (Figure 3B) to the same extent as in the p110δ+iSH2 complex. The changes in deuterium exchange for these regions correlated very well with our activity results: addition of PDGFR pY to the p110δ+nicSH2 complex resulted in activity and deuteration levels similar to that of the p110δ+iSH2 complex. One peptide in the kinase domain, 939–958, only showed decreases in exchange (0.5 Da) when comparing p110δ+nicSH2 to ΔABD-p110δ. This region is located adjacent to the C-terminal helix and may form another contact surface for the cSH2.

### PDGFR pY Increases p110δ/p85α Affinity for Membrane Binding

**Figure 4:**
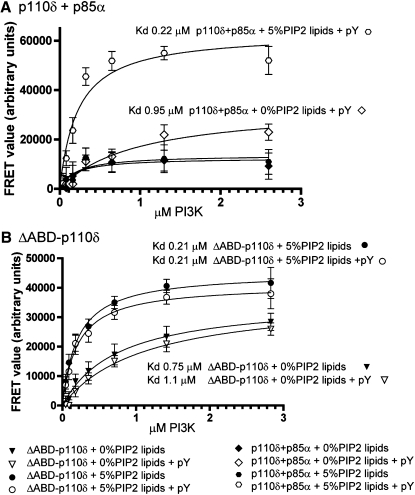
Protein-Lipid FRET Measured from Intrinsic Tryptophanes to the DANSYL Probe of the DANSYL-PS-Containing Liposomes of the Free Catalytic Subunit and Full Length p110δ/p85α Complex in the Presence and Absence of PDGFR pY (A) Lipid binding of the p110δ/p85α complex with 0% and 5% PIP2 lipid vesicles in the presence and absence of PDGFR pY. (B) Lipid binding of the ΔABD-p110δ construct with 0% and 5% PIP2 lipid vesicles in the presence and absence of PDGFR pY. Experiments were repeated in triplicate and graphs are shown ± SD.

**Figure 5:**
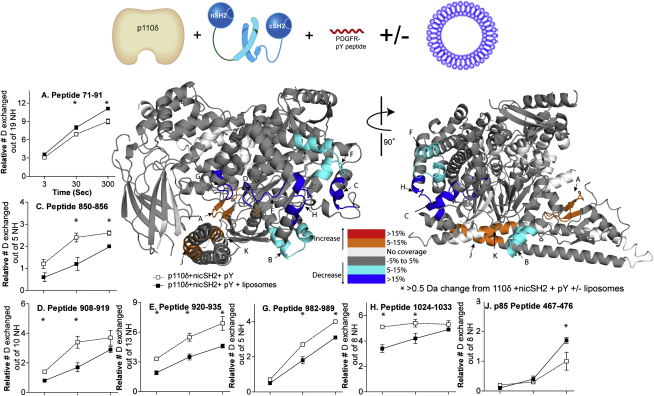
Changes in Deuteration Levels of p110δ and p85α in the Presence of 5% PIP2 Vesicles at 1 mg/ml A model for the iSH2 domain of p85α and ABD domain of the p110δ was generated by combining the ΔABD-p110δ structure (2WXH) with the structure of p110α in complex with niSH2 (3HIZ) (Mandelker et al., 2009). The C-terminal helix of the kinase domain that is disordered in p110δ is modeled (region H) from the structure of p110γ (1E7U) (Walker et al., 1999). Peptides spanning p110δ and p85α (labeled A–K) that showed >0.5 Da changes in deuteration level in the presence of vesicles are mapped onto the model. The percent change mapped on the structure according to the legend is the highest deuterium exchange difference change seen at any time point in the analysis. Peptides that showed a >10% change at any time point are graphed and shown below the figure. Experiments were performed in duplicate, and graphs are shown ± SD. All other peptides with changes >0.5 Da are shown in Figure S5. The graphs are labeled (^∗^) for any time points with a >0.5 Da change between p110δ+nicSH2 + pY in the presence of lipids (see also Figure S5).

To understand the PI3K activation by PDGFR pY binding we examined the affinity of p110δ for lipid membranes using protein-lipid FRET as applied previously to examine Vps34 lipid interactions (Miller et al., 2010). Two different types of lipid vesicles were examined, one with 5% PIP2 present (20% PS/10% Dansyl PS, 35% PE/15% PC/10% cholesterol, 5% sphingomyelin, 5% PIP2), and one with 0% PIP2, where PIP2 was replaced by PS. These experiments showed that the p110δ/p85α complex had very low lipid binding in the absence of PDGFR pY. However, in the presence of PDGFR pY, the p110δ/p85α complex bound PIP2-containing vesicles with much higher affinity (Figure 4). The ΔABD-p110δ construct, which is devoid of any inhibitory contacts imposed by p85 (as well as lacking ABD), bound to PIP2-containing membranes with high affinity, similarly to the PDGFR pY-activated p110δ/p85α (Figure 5). The presence of PDGFR pY had no effect on lipid binding of the ΔABD-p110δ. Binding to lipid vesicles containing 5% PIP2 was more efficient compared to vesicles lacking PIP2, for both the free catalytic subunit and the PDGFR pY-activated p110δ/p85α construct. The PDGFR pY activation of p110δ/p85α also caused a small increase in binding to lipid vesicles lacking PIP2, showing that PIP2 substrate is not the only component involved in PI3K binding to membranes.

### Changes in Exchange Caused by Interaction with Lipid Vesicles

PI3K must access its substrate at the lipid interface, and understanding changes in protein dynamics on binding the lipid interface is important for understanding the mechanism of regulation. Toward this end, we performed deuterium exchange experiments at three time points (3, 30, and 300 s) with a PDGFR pY-activated complex of p110δ+nicSH2 in the presence and absence of 5% PIP2-containing lipid vesicles. These experiments were carried out in the absence of Mg^2+^ and ATP to prevent enzyme activity. Several distinct regions in p110δ (698–715, 850–856, 908–919, 920–935, 939–958, 982–989, and 1024–1033) and p85α (414–420) had decreases in exchange in the presence of lipid substrate (Figure 5; Figure S5). Regions 698–715 spanning the loop between kα1 and kα2 in the kinase domain and 850–856 covering kα5 and the loop between kα4 and kα5 were both previously proposed to participate in membrane binding (Huang et al., 2007; Mandelker et al., 2009; Walker et al., 1999). Region 908–919 containing the beginning of the substrate binding loop (encompassing the DFG motif) also had a decrease in exchange in the presence of lipid vesicles. Region 982–989 is adjacent to the substrate binding loop in the structure. It contains part of kα9 and the loop connecting to kα10. The p110α helix equivalent to p110δ helix kα10 is known to interact with the nSH2 domain (Mandelker et al., 2009). Region 1024–1033 at the C-terminal end of p110δ is equivalent to the C-terminal tail of class III PI3Ks that was shown to bind membranes (Miller et al., 2010). Interestingly, regions 920–935, 939–958, and 1024–1033 were all protected by the presence of the nicSH2 and also all show an interaction with lipid vesicles, suggesting that p85 greatly regulates the interaction of p110 with membranes.

Surprisingly, we find some regions that have an increase in exchange in the presence of lipid vesicles. One is in p110δ, region 71–91, which is in the ABD domain, and the other two are in the iSH2 domain of p85α (regions 467–476 and 556–570). This indicates that the presence of lipid vesicles may loosen the interaction between the iSH2 and the p110δ. Experiments were also performed on the full length p110δ/p85α complex in the presence of PDGFR pY and 10 mM Mg^2+^ with and without 5 mM nonhydrolysable ATP analog, AMPPNP to examine possible changes on nucleotide binding. No changes were seen in the catalytic subunit on AMPPNP/Mg^2+^ binding (data not shown). This is not surprising because peptides spanning the ATP binding pocket (752–762, 886–900) experience <25% exchange even at 3000 s.

### Engineering p85α Mutations that Activate p110δ

**Figure 6:**
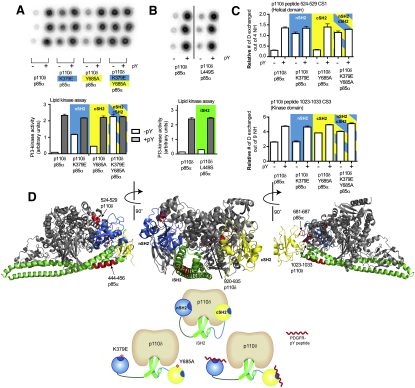
Effect of the p85α K379E and Y685A Mutations on Lipid Kinase Activity and Deuterium Exchange in Vitro with a Model of p110δ/p85α Regulatory Interaction (A) PI3K activity of p110δ, with full length p85α constructs containing nSH2 (K379E) and cSH2 (Y685A) mutations in the presence and absence of PDGFR pY. Assays measured ^32^P-PIP3 production in the presence of 10 nM enzyme, 100 μM ATP and 5% PIP2 lipid vesicles at a concentration of 1 mg/ml. PDGFR pY was 10 μM. (B) PI3K activity of p110δ, with full-length p85α containing the cancer-linked L449S iSH2 mutation in the presence and absence of PDGFR pY. All lipid kinase activity assays were performed in triplicate and graphs are shown ± SD. (C) The deuteration level at 1000 s of on-exchange for a helical domain peptide (524–529), and at 3 s of on exchange for a C-terminal peptide (1023–1033) was plotted for eight conditions as indicated on the legend. Experiments were performed in duplicate, and graphs are shown ± SD. (D) A structural model for the interaction of p110δ with the nSH2, iSH2, and cSH2 domains of p85α was generated using the crystal structure of the free p110δ catalytic subunit (2WXH), with the nSH2 from the p110α/p85α structure (3HHM), the iSH2 (2VIY), and the cSH2 from the recent p110β/p85β structure (2Y3A). Regions with changes on phosphopeptide binding are colored in red and labeled on the model (see also Figure S6).

DXMS identified the region from 681–687 in the cSH2 as part of the interface with p110δ. We hypothesized that the cSH2 may be responsible for the observed changes in exposure of the C-terminal end of the p110δ kinase domain when p110δ is bound to nicSH2 compared to free p110δ. To test this hypothesis, we mutated Tyr-685 in the p85α cSH2 to alanine. The p110δ+nicSH2 Y685A mutant complex showed higher basal activity than the equivalent wild-type complex and the same basal activity as the p110δ+niSH2 complex. In the presence of PDGFR pY, the mutant complex was activated to a similar level as the niSH2 and nicSH2 complexes (Figure 1C). To examine if this mutation was functionally important in the regulation of the full length p110δ/p85α complex, as well as to determine individual effects of the nSH2 and cSH2, we purified full length complexes containing the K379E nSH2 mutation, the Y685A cSH2 mutation, and the double mutant K379E, Y685A. The engineered oncogenic K379E mutation in nSH2 has been previously shown to activate p110α both in vitro and in vivo (Miled et al., 2007; Sun et al., 2010) by breaking the contact between the nSH2 and the helical domain. Enzyme activity assays performed with 5% PIP2 liposomes showed that the K379E or Y685A p85 mutations activate the enzyme 10-fold and 5-fold, respectively (Figure 6A), and that the presence of both mutations fully activated the enzyme, and made it insensitive to further PDGFR pY activation.

Deuterium exchange experiments were then carried out on the K379E, Y685A, and double mutant complexes. Both of these mutations maintained the ability to bind PDGFR pY as shown by similar decreases in exchange in peptide 414–420 of the nSH2 and 704–710 of the cSH2 in the presence of PDGFR pY (Figure S6). The presence of the nSH2 mutation (K379E) exposed the p110δ helical domain peptide 524–529 to a similar level as when in the presence of PDGFR pY, whereas the Y685A mutation exposed the elbow peptide 1023–1033 of the p110δ kinase domain (Figure 6C), as well as the substrate binding loop peptide 920–935 (Figure S6). This C-terminal elbow showed a 1.0 Da increase in exchange at 3 s of on-exchange in the Y685A mutant complex compared with wild-type, and showed a further 1.0 Da increase in the presence of PDGFR pY (Figure 6C). The peptide 681–687 in p85α that showed an increase in exchange in the presence of PDGFR pY in wild-type p85α, showed a decrease in exchange for the Y685A construct due to rigidification of the entire cSH2 domain in the presence of PDGFR pY (Figure S6). This is further evidence that this p85 region represents a key contact site with the catalytic subunit, and that the presence of the Y685A mutation in the cSH2 does not disrupt PDGFR pY binding. The peptide 444–456 in the iSH2 domain of p85α showed a 1.0 Da increase in exchange at 1000 s in the double mutant complex compared to wild-type and was further exposed by the addition of PDGFR pY (Figure S6). To test the possible effect of mutations in this region on enzyme activity we expressed the full length complex of p110δ/p85α with the previously determined p85α oncogenic mutation L449S (Jaiswal et al., 2009) and found that this mutation increased basal activity ∼2-fold and showed activation by PDGFR pY that was similar to the wild-type (Figure 6B).

**Figure 7:**
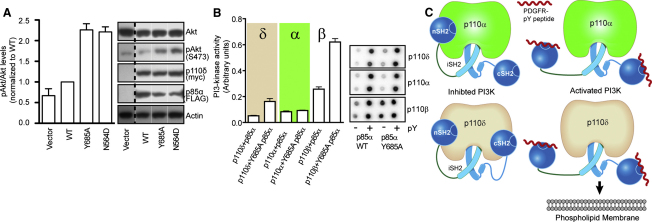
Effect of the p85α Y685A Mutation on Lipid Kinase Activity In Vitro and PI3K Signaling in Cell Culture with a Model of p110δ/p85α Regulatory Interaction (A) Y685A mutation in the cSH2 of p85α (Y685A) increases Akt phosphorylation (Ser473) in HEK cells overexpressing p110δ+p85α heterodimers. Bar graphs show mean ± SEM (n = 3) of phosphorylated Akt (pAkt) to Akt ratios normalized to wild-type p110δ+p85α (WT). (B) PI3K activity of p110δ, p110α, and p110β in the presence of full length p85α (WT and Y685A) in the presence and absence of PDGFR pY. Assays measured ^32^P-PIP3 production in the presence of 10 nM enzyme, 100 μM ATP and 5% PIP2 lipid vesicles at a concentration of 1 mg/ml. PDGFR pY was 10 μM. PI3K assays were performed in duplicate and repeated three times. Shown is a representative experiment with graphs shown ± SD. (C) A model for the regulation of p110δ and p110α basal activity by the nicSH2 domains of p85α and activation by PDGFR pY. The presence of the nSH2 and cSH2 domains prevents lipid binding, and binding of PDGFR pY exposes lipid interacting regions and increases membrane affinity.

In addition, cellular assays looking at PI3K downstream signaling showed a 2-fold increase in Akt phosphorylation for cells transiently cotransfected with the p110δ+p85α Y685A compared to the cells cotransfected with p110δ+p85α (Figure 7A). A p110δ +p85α N564D complex, carrying a somatic mutation in the iSH2 previously demonstrated to potently activate PI3K signaling, resulting in oncogenesis (Jaiswal et al., 2009), showed a similar increase in Akt phosphorylation (Figure 7A).

To determine if there were any p110 isoform specific differences in regulation by cSH2, we purified full-length complexes of human p110α, p110β, and p110δ with and without the Y685A mutation in p85α. Enzyme activity assays performed with 5% PIP2 liposomes showed that both p110δ and p110β were activated by the mutation, whereas p110α was not (Figure 7B).

## Discussion

Understanding the regulation of different isoforms of the class IA PI3Ks is an important goal due to their prominent and isotype-specific roles in human diseases including cancer, diabetes, inflammation, thrombosis, allergy, and cardiac disease. In the absence of activators such as phosphorylated RTKs, the basal activity of p110δ is very low, kept tightly suppressed by p85. Understanding the dynamic interactions between the catalytic and regulatory subunits is important in defining the mechanism of PI3K regulation. The crystal structure of p110α with the niSH2 construct of p85α shows contacts formed between the two subunits (Mandelker et al., 2009), but it does not explain how these contacts translate into inhibition of the enzyme. DXMS for p110δ/p85α has allowed us to interrogate how contacts between the regulatory and catalytic subunits cause dynamic changes throughout the enzyme.

The presence of the iSH2 caused decreases in exchange all along the crevice of the p110δ where the iSH2 is predicted to bind (based on the crystal structures of p110α and p110β complexes) (Mandelker et al., 2009; Zhang et al., 2011). One of the regions in the C2 domain with decreased exchange in the presence of the iSH2 contains Asn334, equivalent to Asn345 in p110α. This is consistent with the published data showing that the interaction between this residue and Asp560/Asn564 in the iSH2 participates in the inhibition of p110α (Wu et al., 2009). The presence of the nicSH2 construct caused decreases in exchange at a region in the helical domain, showing that the interaction with the nSH2 previously demonstrated for p110α is conserved in p110δ (Mandelker et al., 2009). Decreases were also seen at the C-terminal elbow following helix kα11, which we predict forms an interface with the cSH2. We showed that the p110δ C-terminal region has a critical role in catalysis, because a deletion construct missing the last 17 residues (from 1028 to 1044) had undetectable lipid kinase activity toward PIP2 containing liposomes. The C-terminal end of the substrate-binding (activation) loop also showed decreases in exchange in the presence of various p85α constructs, and it is interesting that the more protected this region was, the lower the lipid kinase activity. This region would not be predicted to be in contact with the cSH2, and is located directly underneath helix kα11 and may represent an allosteric change on rigidification of helix kα11. The same regions in p110δ that had decreases in exchange with the nSH2 and cSH2 showed an increase in the presence of PDGFR pY, restoring the same level of exchange observed with only the iSH2. These observations indicate that breaking these contacts disinhibits the enzyme.

We find that p110δ/p85α disinhibition correlates with increased enzyme affinity for membrane vesicles: the p110δ/p85α complex binds membranes very poorly and the addition of PDGFR pY greatly increases the affinity of the p110δ/p85α for PIP2-containing membranes and to a lesser extent also to membranes lacking PIP2. The binding of the PDGFR pY-activated p110δ/p85α to membranes is similar to the binding of the free catalytic subunit, ΔABD-p110δ that is devoid completely of p85 inhibitory contacts. This surprising result that the free catalytic subunit can bind lipid as effectively as the activated p110δ/p85α complex contrasts with the hypothesized role of the iSH2 domain in lipid binding (Huang et al., 2007). Regions exposed in the kinase domain on PDGFR pY binding had decreases in exchange in the presence of lipid substrate, revealing that PDGFR pY binding is critical in exposing regions of the protein that interact with the lipid membrane. We expected to see changes in the C2 domain due to lipid interaction (Denley et al., 2008), however, we see no significant changes, but no peptic peptides covering the CBR3 region of the C2 domain were identified and analyzed.

In order to generate a regulatory subunit that would selectively disrupt the contact between the cSH2 and catalytic domains, we made a Y685A p85α mutant based on our finding that the peptide containing this residue becomes exposed on PDGFR pY binding. This mutation caused an increase in basal activity of p110δ, increased downstream signaling in cells and led to exposure of the C-terminal end of the C-lobe and substrate-binding loop, while not affecting exchange at the helical domain nor the PDGFR pY binding site. This engineered mutation in p85α also activated recombinant p110β but did not activate p110α, revealing isoform specific aspects of regulation. This mutation has been recently found to activate p110β in a complex with p85β as well, and the crystal structure of p110β with the icSH2 construct of p85β shows that this Tyr residue is located at the center of the interface between the catalytic and regulatory subunit (Zhang et al., 2011). The presence of a previously identified engineered oncogenic nSH2 mutation (K379E) alone increased the lipid kinase activity and the presence of the double mutant (K379E, Y685A) in p85α fully activated the enzyme as well as exposing similar regions to exchange as in the PDGFR pY activated complex (Figure 6D). These p85 point mutations allowed us to isolate specific effects of the nSH2 and cSH2 domains on the dynamics of the p110δ subunit.

Based on our results, and using the previously solved structures of p110α with the niSH2 construct of p85α (Mandelker et al., 2009) and p110β with the icSH2 construct of p85β (Zhang et al., 2011) we have constructed a model for the regulation of p110δ by the nicSH2 domains of p85α (Figure 6D). We propose that both the nSH2 and cSH2 play important roles in inhibiting the kinase activity of p110δ, in contrast to p110α, which is inhibited only by the nSH2 (Figure 7C). The nSH2 and cSH2 domains prevent binding of p110δ/p85α to membranes. Binding of PDGFR pY breaks inhibitory contacts formed by the nSH2 and cSH2 with p110δ and increases affinity for membrane vesicles.

Several oncogenic mutations in p85α have been recently identified and analyzed (Jaiswal et al., 2009; Sun et al., 2010; Wu et al., 2009). Two of them map to regions in the p85 where we see increases in exchange in the presence of PDGFR pY (L449S in the iSH2 and A682V in the cSH2). We have shown that the L449S mutation activates the basal enzyme activity. The presence of the A682V mutation, which is located in the proposed interface of the catalytic subunit with the cSH2, may contribute to relieving the inhibitory cSH2 contact. Many of the iSH2 mutations are located in or closely adjacent to regions where we see an increase in exchange in the presence of lipid vesicles (D464H, D560Y, N564K/D, and L570P). Because we show that interaction with lipid vesicles causes a loosening between the iSH2 and p110δ, these mutations may mimic this membrane-mediated loosening and activate by disrupting the interaction between iSH2 and p110, as previously proposed (Wu et al., 2009).

This is the first study to examine the dynamic interactions of heterodimeric complexes containing the full-length class IA catalytic subunit with its regulatory subunit, and show how the addition of PDGFR pY as well as lipid vesicles affects these interactions. Based on these, we have identified a novel isoform-specific regulatory function for the cSH2 of p85α with p110δ. For the first time, we have shown that RTK pY peptide binding directly increases membrane binding of the class IA heterodimer. We mapped multiple regions of an activated PI3K that interact with membranes and provide experimental evidence for the dynamic interaction with lipids. The gain in exposure in some regions in the presence of membranes indicates that loosening of interactions between the p110δ/p85α complex accompanies catalysis on membranes. Our DXMS results provide a framework for designing further structural and biochemical approaches to understand regulation of the PI3K and PI3K like enzyme families.

## Experimental Procedures

### Protein Expression and Kinase Assays

All protein constructs were expressed in Sf9 cells as described for the ΔABD-p110δ (Berndt et al., 2010). Full details are described in the Supplemental Experimental Procedures.

### Lipid Kinase Assays

The lipid kinase activity was determined using a modified membrane capture assay measuring production of ^32^P-labeled PIP3 (Knight et al., 2007). Lipid vesicles were used at a final concentration of 1 mg/ml and were composed of 5% brain PIP2, 20% brain PS, 45% brain PE, 15% brain PC, 10% cholesterol, and 5% sphingomyelin (Avanti Polar Lipids). Vesicle preparation is described in Supplemental Experimental Procedures. The lipid solution was mixed with each protein construct, with a final buffer containing 3 mM MgCl_2_, 1 mM EGTA, 20 mM Tris pH-7.5, 50 mM NaCl, and 50 mM KCl. Reactions were started by adding 100 μM ATP (final concentration) containing 0.1 μCi per μL of [γ^32^P]-ATP in a total volume of 15 μL. This reaction was carried out for sixty minutes, and was stopped by mixing 3 μl of the reaction mixture with 3 μl of 20 mM EDTA. Three microliters of this mixture was then spotted on a nitrocellulose membrane. The membrane was washed six times with a 1 M NaCl/1% phosphoric acid solution. After all washes the membrane was dried for one hour, followed by a 5–20 min exposure to a phosphor screen (Molecular Dynamics). The spot intensity on the phosphor screen was imaged using a Typhoon phosphoimager (GE Healthcare). The intensity was quantitated using ImageQuant (GE Healthcare).

### Deuterium Exchange Measurements

Stock solutions of protein (30 pmol/μl) were prepared in 20 mM Tris pH-7.2, 50 mM ammonium sulfate, 1% ethylene glycol, and 5 mM DTT following the procedure in the Supplemental Experimental Procedures. Measurements in the presence of PDGFR pY were carried out by a 5-fold dilution of a 1 mM PDGFR pY (mouse PDGFR residues 735–767, peptide sequence ESDGG(pY)MDMSKDESID(pY)VPMLDMKGDIKYADIE) stock in 10 mM HEPES pH-7.2, 2% DMSO giving a final concentration of 200 μM PDGFR pY before addition of deuterium. Exchange reactions were initiated by the addition of 4 μl of protein stock to 1 μl of either PDGFR pY solution or blank and allowed to equilibrate for 10 min followed by the addition of 25 μl of a 98% D_2_O solution containing 10 mM HEPES pD-7.2, 50 mM NaCl, and 2 mM DTT, giving a final concentration of 82% D_2_O. Deuterium exchange reactions were allowed to carry on for five time points, 3, 10, 100, 1000, and 3000 s of on-exchange at 23°C, before addition of quench buffer. For 3 s of on-exchange, an experiment was also performed at 0°C to examine exchange rates of extremely rapidly exchanging amide hydrogens. On-exchange was stopped by addition of 40 μl of quench buffer containing 1.2% formic acid and 1.66 M Guanidine-HCl, which lowered the pH to 2.6. Samples were then immediately frozen in liquid nitrogen until mass analysis. Every time point and condition was a unique experiment, and every DXMS experiment was repeated in duplicate.

For DXMS studies on the K379E, Y685A, and double mutants, on-exchange experiments were carried out on the full length complexes of p110δ and p85α at two time points (3 and 1000 s) that between them covered all time points with differences in exchange on nicSH2 binding.

For lipid binding experiments, lipid vesicles were prepared at 5 mg/ml as described in Lipid Kinase Assays and were diluted 5-fold with a 98% D_2_O solution containing 10 mM HEPES pD-7.2, 50 mM NaCl, and 2 mM DTT. Deuterium exchange reactions were started by mixing 5 μl of a protein stock solution (15 pmol/μl) with 25 μl of deuterated lipid buffer (25 μg phospholipid) giving a final solution of 65% D_2_O. Half as much protein was used in lipid binding experiments to maximize the ratio of lipid to protein in the sample. Higher concentrations of protein or lipid in the sample led to sample aggregation. For this reason there was no chance to preincubate the protein solution with lipid vesicles before addition of D_2_O due to aggregation of protein when combined with high levels of lipid vesicles.

For AMPPNP binding experiments, protein was incubated with 10 mM Mg^2+^ and +/− 5 mM AMPPNP for 15 min and then 5 μl of the protein/nucleotide solution was diluted with 25 μl of deuterated buffer. All other steps remained the same.

Full experimental procedures are described in the Supplemental Experimental Procedures.

## Supplemental Information

Document S1. Seven Figures, One Table, and Supplemental Experimental Procedures
