# Supplementary material for: Dynamics of the Phosphoinositide 3-Kinase p110δ Interaction with p85α and Membranes Reveals Aspects of Regulation Distinct from p110α
Source: Structure. 2011 Aug 10;19(8):1127–37. doi: 10.1016/j.str.2011.06.003 (PMC3155019; doi:10.1016/j.str.2011.06.003)
Supplement: Document S1. Seven Figures, One Table, and Supplemental Experimental Procedures [file mmc1.pdf]

Structure *19*

Supplemental Information

Dynamics of the Phosphoinositide 3-Kinase p110δ Interaction with p85α and Membranes Reveals Aspects of Regulation Distinct from p110α

John E. Burke, Oscar Vadas, Alex Berndt, Tara Finegan, Olga Perisic, and Roger L. Williams

Fig. S1. PI3K domains and peptide coverage after pepsin digestion. A. Constructs purified and examined are shown with sequence numbers corresponding to the p110δ gene sequence, and p85α gene sequence. B. Pepsin digested peptide coverage map of p110δ and p85α. Identified and analyzed pepsin-digested peptides are shown under the primary sequence of p110δ and p85α. The primary sequences of p110δ and p85α are colored according to domain boundaries shown above. Gray bars indicate peptides continued in the next line of the sequence.

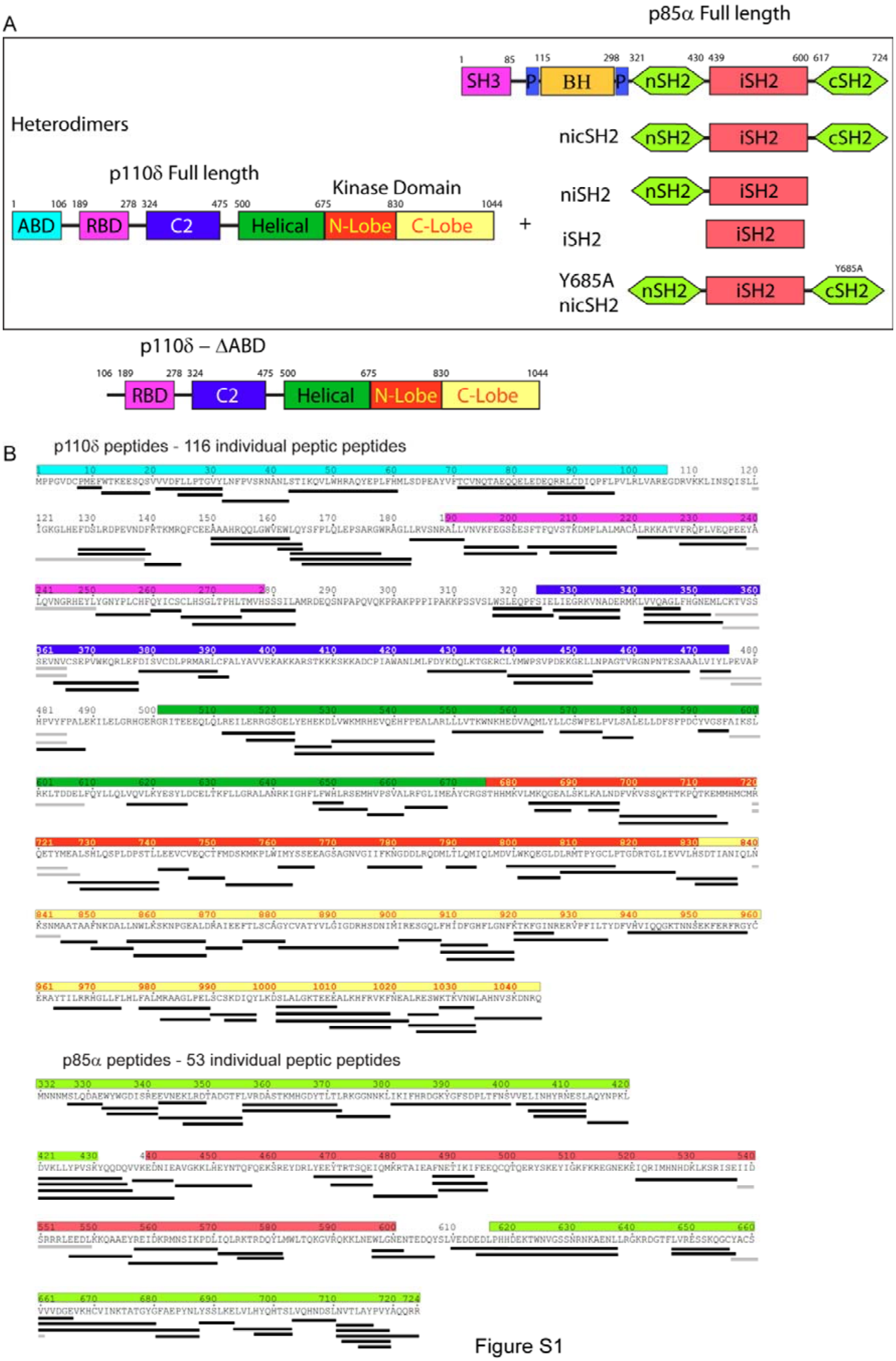
A
p85α Full length
SH3
P
BH
P
nSH2
iSH2
cSH2
Heterodimers
nicSH2
niSH2
iSH2
Y685A
nicSH2
p110δ Full length
Kinase Domain
ABD
RBD
C2
Helical
N-Lobe
C-Lobe
+
p110δ – ΔABD
B
p110δ peptides - 116 individual peptic peptides
p85α peptides - 53 individual peptic peptides

Figure S1

Fig. S2. Global exchange of ΔABD-p110δ and p85α nicSH2. (**A**) The relative deuteration levels for the ΔABD-p110δ construct at three time points (3, 100 and 3000 seconds) were mapped onto the crystal structure of this construct (2WXH). The color of the protein indicates relative deuterium levels. Areas of the protein with no peptide coverage are colored gray. (**B**) The relative deuteration levels of the p85α nicSH2 construct (residues 322-724) in the context of complex with the full-length p110δ are mapped onto three isolated crystal structures (2VIY for the iSH2, 2IUI for the nSH2, and 1H9O for the cSH2). The deuterium exchange rates correlated well to the crystal structure, with interior regions protected from exchange, and unstructured regions, surface loops, and regions with high crystallographic B factors having the highest exchange rates. The fastest exchanging peptides of the protein spanned the surface loop from 840-850, which was proposed to be one of the loops important for membrane binding in p110α (Mandelker et al., 2009). Peptides covering the C-terminus of the protein, residues 1023-1044, and residues 920-926 from the substrate-binding loop, a critical loop for lipid substrate recognition (Bondeva et al., 1998) (also known as the activation loop by analogy with protein kinases), were also fully deuterated after only 3 seconds of on-exchange at 23 °C. This part of the substrate-binding loop is disordered in the p110δ crystal structure (Berndt et al., 2010). Peptides covering the beginning of the substrate-binding loop from 908-919, which contain the DFG motif, were protected from exchange at early time points, but were almost fully deuterated at 3000 seconds of on-exchange. Peptides spanning the catalytic loop from 887-898, and a region of the helical domain from 530-564, remained protected from deuteration at all time points, indicating a rigid secondary structure or lack of solvent accessibility. The peptides spanning the iSH2 coiled coil domain from 467-570 showed very slow exchange rates at all time points. This is consistent with this domain serving as a rigid scaffold for p110 binding (Fu et al., 2004). However, peptides spanning the N-terminal and C-terminal ends from 436-443 and 582-606 of the iSH2 domain are both over 40% deuterated after 1000 seconds of on-exchange. The nSH2 and cSH2 domains showed similar rates of exchange to each other, as expected from the similarity of structures solved by both NMR and crystallography.

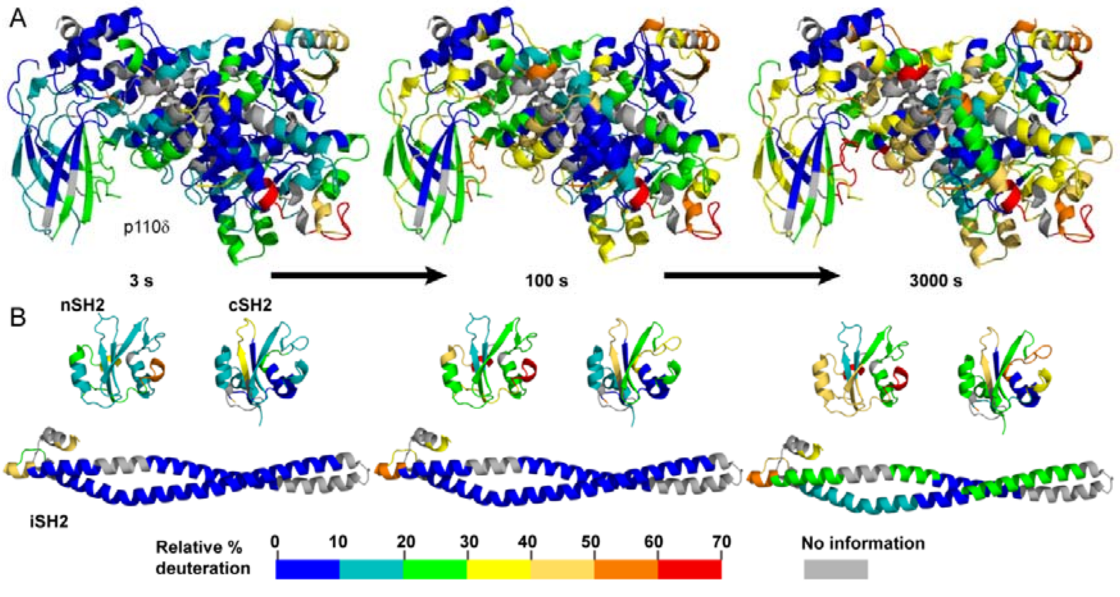

Fig. S2

Fig. S3. Changes in deuteration levels of the p85α nicSH$_2$ construct in the presence and absence of PDGFR phosphopeptide (complement to main figure 2). Peptides spanning the p85α regulatory

subunit that showed greater than 0.5 Da changes in deuteration level in the presence and absence of the nicSH$_2$ construct of p85α are colored onto the structures (2VIY, 2IUI, and 1H9O) according to the legend. The structures of phosphopeptide bound to the cSH2 and nSH2 domains are colored purple in the figure. Peptides are labeled A-M. The graphs are labeled (*) for any time point with a greater than 0.5 Da changes between the p110δ/ p85α nicSH$_2$ +/- PDGFR phosphopeptide.

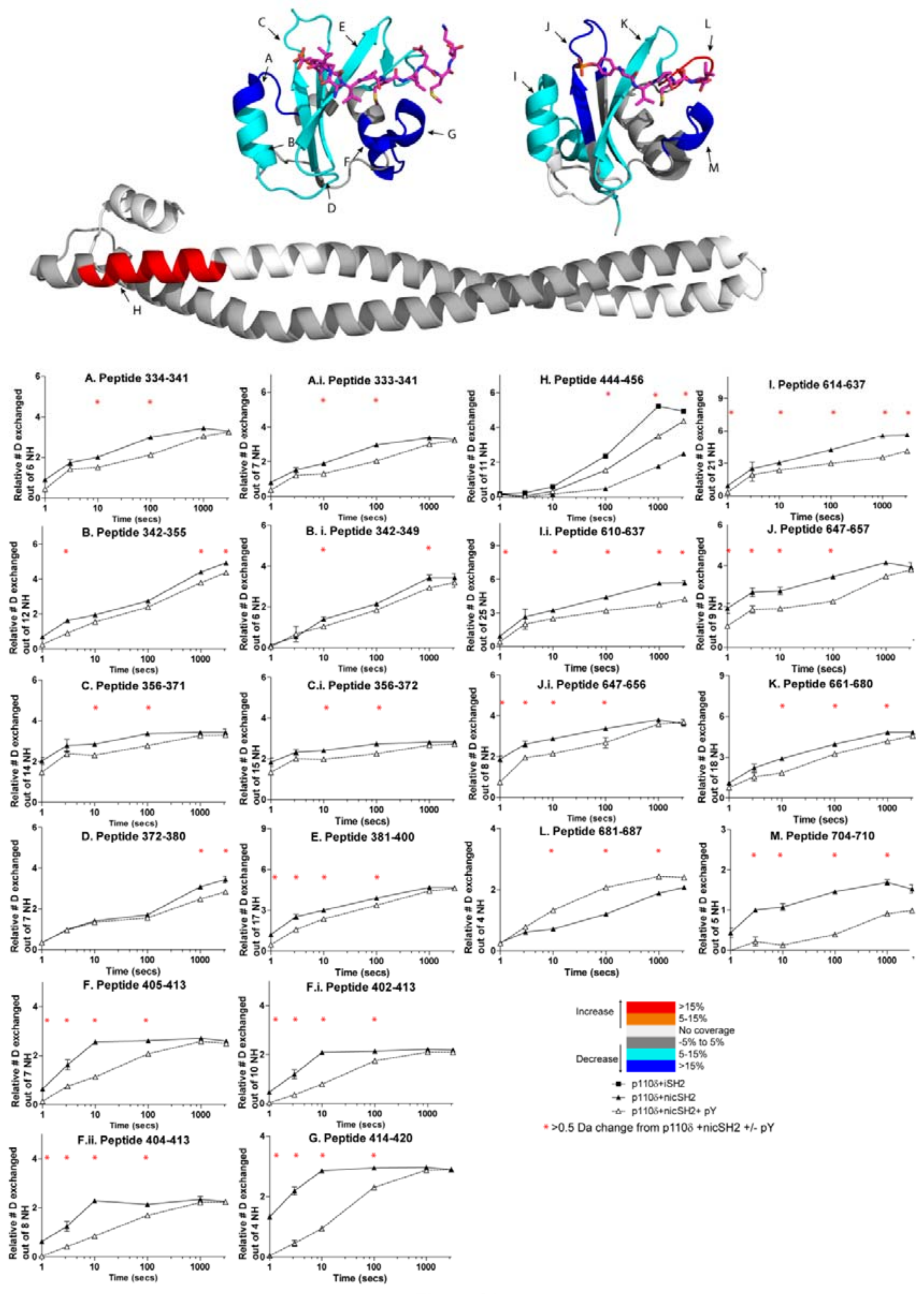

Figure S3

Fig. S4. Changes in deuteration levels of the free catalytic domain in the presence of both nicSH$_2$ p85α and PDGFR phosphopetide (complement to main figure 3). A. Peptides spanning the p110δ catalytic subunit that showed greater than 0.5 Da changes in deuteration level in the presence and

absence of the $nicSH_2$ construct of p85α are colored onto the structure (2WXH) according to the legend. Peptides are labeled A-I. B. Peptides spanning the p110δ catalytic subunit that showed greater than 0.5 Da changes in deuteration level in the presence of $nicSH_2$ +/- a PDGFR phosphopeptide are colored onto the structure according to the legend. The graphs are labeled (*) for any time points with a greater than 0.5 Da change between the ΔABD- p110δ and p110δ + $nicSH_2$ p85α constructs, and labeled (*) for any time point with a greater than 0.5 Da changes between the p110δ/ p85α $nicSH_2$ +/- PDGFR phosphopeptide.

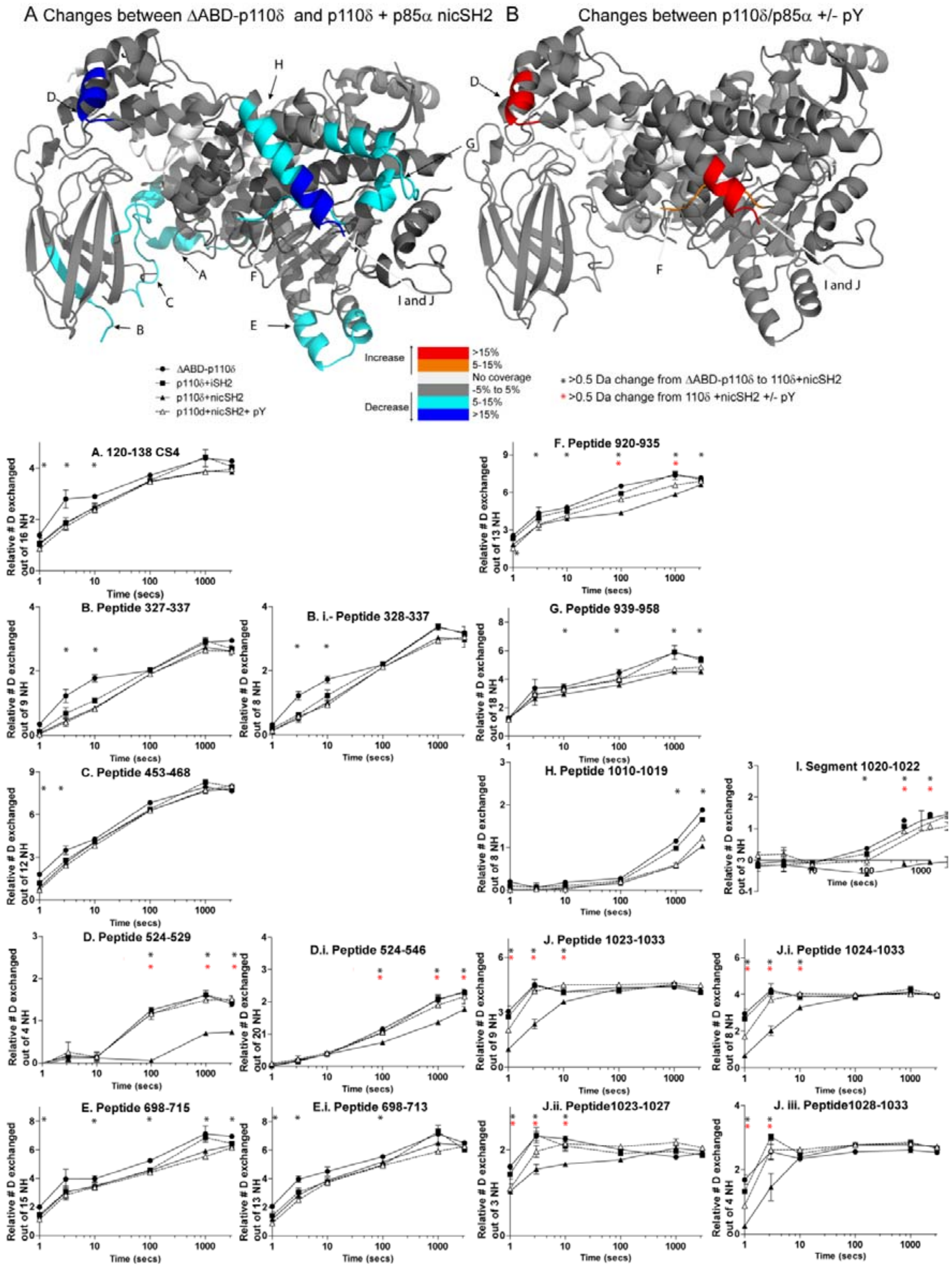

Figure S4

Fig. S5. Changes in deuteration levels of p110δ catalytic subunit and p85α regulatory subunit in the presence of lipid vesicles (complement to main figure 5). A model containing the iSH2 domain of p85α and ABD domain of the catalytic subunit was generated by combining the ΔABD-p110δ

structure (2WXH) with the recently solved structure of p110α in complex with niSH2 (3HIZ) (Mandelker et al., 2009). The c-terminal helix of the kinase domain that is disordered in p110δ is modeled (region H) from the structure of p110γ (1E7U) (Walker et al., 1999) (A) Peptides spanning the p110δ catalytic subunit and the p85α regulatory subunit and that showed greater than 0.5 Da changes in deuteration level in the presence of lipid vesicles are mapped onto the model according to the legend. Peptides are labeled A-K. All experiments were performed in duplicate. The graphs are labeled (*) for any time points with a greater than 0.5 Da change between p110δ+nicSH2 + pY in the presence of 1mg/ml of 5% PIP2 lipid vesicles.

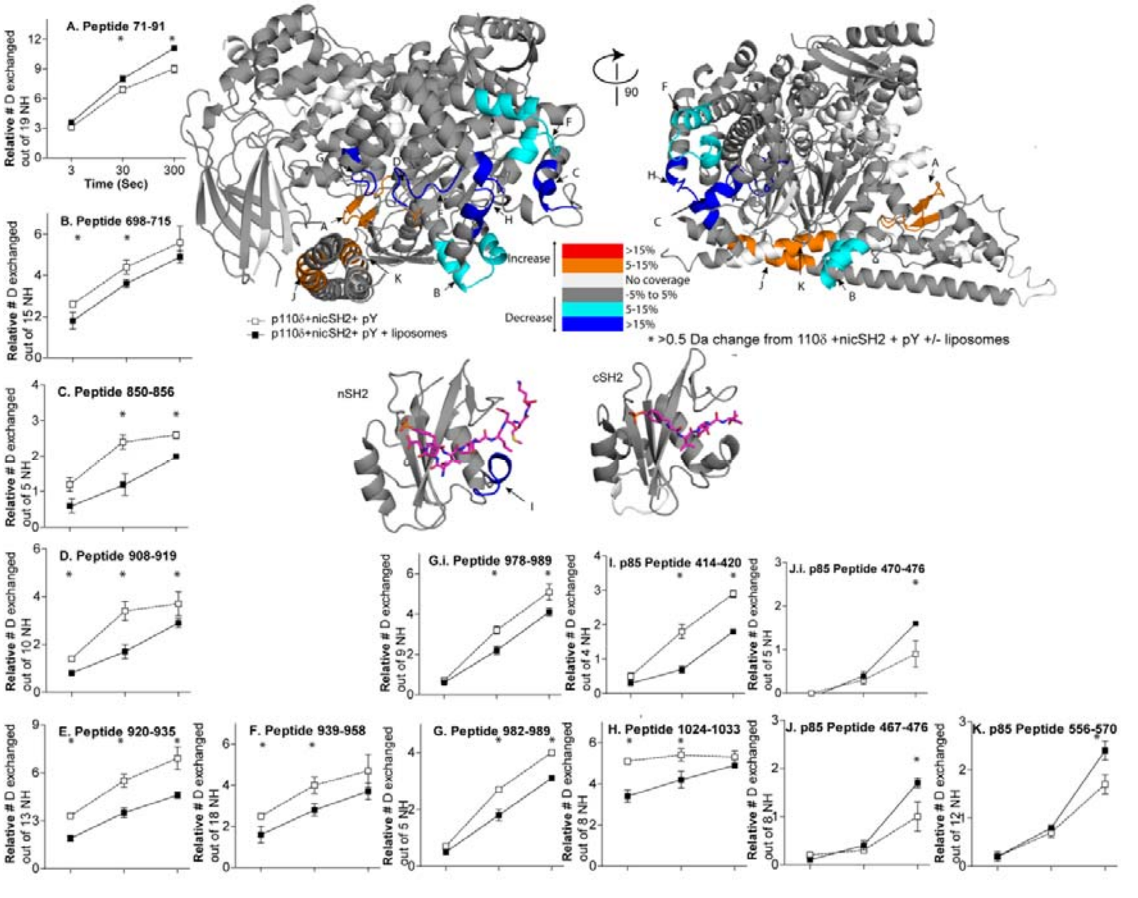

Figure S5

Fig. S6. Changes in deuterium exchange caused by K379E and Y685A mutations in p85α nSH2 and cSH2 (complement to main figure 6). A. The relative deuterium level of peptides 524-529 and 920-935 of p110δ, and peptides 444-456, and 704-710 of p85α are shown for various conditions at 1000 seconds of on exchange. The relative deuterium level of p110δ peptide 1023-1033 and p85α peptide 414-420 for various conditions at 3 seconds of on exchange is also shown. This mutants were correctly folded as shown by their ability to still bind RTK pY peptide, as indicated by similar

decreases in exchange in the nSH2 (414-420) and cSH2 domains (704-710) for the mutant and wild type complexes. Changes in deuterium exchange for K379E and Y685A p85 constructs are shown for peptides 681-687 and 373-380 at 3 seconds of on exchange. An asterisk identifies that these experiments are not comparing the same peptide, for every condition. Mutant complexes contain peptides with different m/z and retention times due to engineered mutations in the sequence. B. A structural model of the interaction of p110δ with the nSH2, iSH2 and cSH2 domains of p85α was generated using the crystal structure of the free p110δ catalytic subunit (2WXH), with the nSH2 from the recent p110α/p85α structure (3HHM), the iSH2 (2VIY), and the cSH2 from the recent p110β/p85β structure (2Y3A). Regions with changes upon phosphopeptide binding are colored in red and labeled on the model.

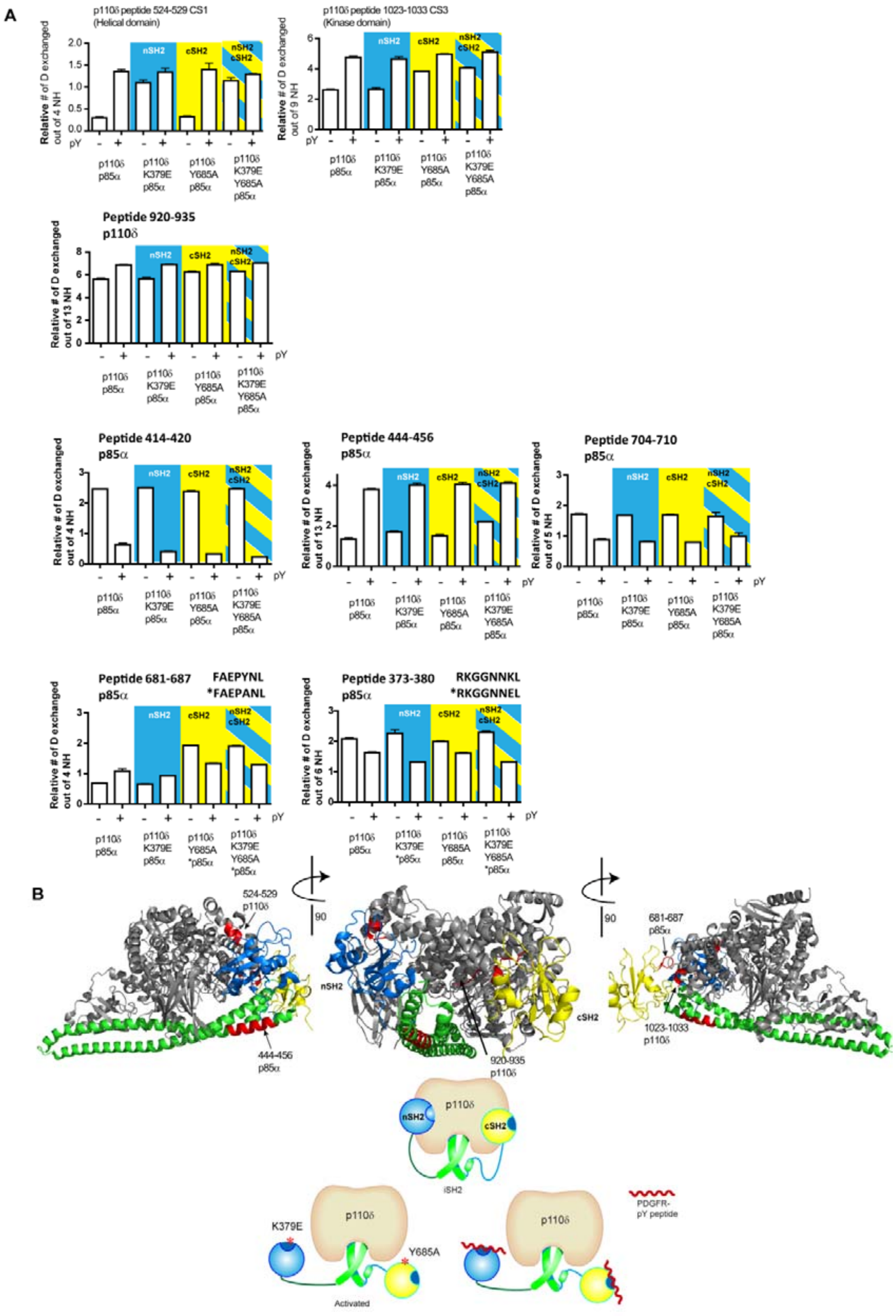

Figure S6

Fig. S7. MS Data processing methodology A. Representative UPLC - MS trace of the p110δ FL + nicSH$_2$ p85α construct peptide digest with pY peptide. B. Representative UPLC - MS trace of the

p110δ FL + nicSH_2 p85α construct peptide digest with pY peptide and phospholipid. No changes in UPLC performance were caused by the presence of phosholipid. C. Shown is the raw data for a selected peptide (1023-1033 charge state 3) that exhibits changes in the presence of nicSH_2 and phosphopeptide. Three different conditions are shown at three different time points (3 seconds on exchange at 0 ^{0}C, and 3 and 10 seconds of on exchange at 23 ^{0}C). The arrow represents the centroid mass calculated by the DXMS software (Sierra analytics). D. The fully processed data is shown for peptide 1023-1033 and peptide 524-529. The 3 seconds of on exchange at 0 ^{0}C is graphed as 1 second of on exchange to fit all data on the same graph. Error bars are calculated as the standard deviation of two separate independent experiments. These two peptides show very different deuterium exchange rate profiles. The shape of deuterium incorporation curves contains important structural information. In this example, peptide 1023-1033 covers an extremely flexible region that shows ~50% relative exchange (the real value will be 20-30% greater due to back exchange) in the ΔABD- p110δ after 3 seconds. The presence of the nicSH2 construct of p85α causes a decrease in exchange, and this decrease is reversible with pY peptide. However, this region is ~50% exchanged after 10 seconds of on-exchange, even in the presence of the nicSH2 construct indicating that this region remains highly flexible and solvent exposed relative to other regions in the protein. Peptide 524-529 represents a different situation where the peptide is completely protected in all conditions through 10 seconds of on exchange. After this time constructs lacking the nSH2 or cSH2 show a progressive incorporation of deuterium up to ~40-50% at 1000 seconds. The presence of the nicSH2 construct prevents all exchange through 100 seconds and this decrease is reversible with pY peptide, but after this time deuterium incorporation begins. However even at 3000 seconds there is still significant protection by the nicSH2 compared to the free subunit. This indicates that this region is conformationally rigid or solvent protected in the p85α bound state, and that the removal of this contact by removal of the SH2 domains or pY peptide binding exposes this region; however the region remains relatively rigid compared to a region like 1023-1033.

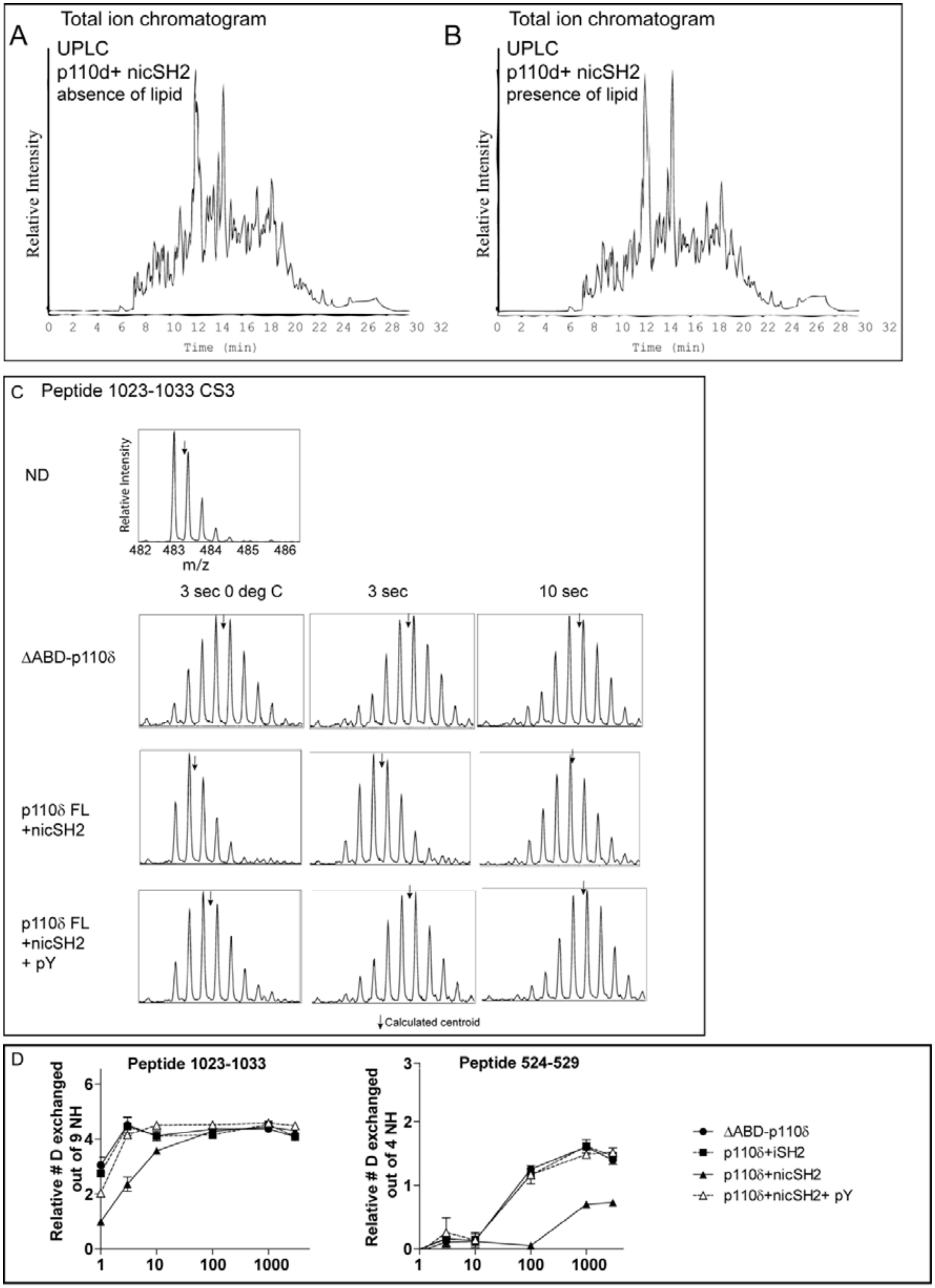

A
Total ion chromatogram
UPLC
p110d+ nicSH2
absence of lipid
Relative Intensity
Time (min)
B
Total ion chromatogram
UPLC
p110d+ nicSH2
presence of lipid
C Peptide 1023-1033 CS3
ND
Relative Intensity
m/z
3 sec 0 deg C
3 sec
10 sec
ΔABD-p110δ
p110δ FL +nicSH2
p110δ FL +nicSH2 + pY
Calculated centroid
D
Peptide 1023-1033
Relative # D exchanged out of 9 NH
Peptide 524-529
Relative # D exchanged out of 4 NH
ΔABD-p110δ
p110δ+iSH2
p110δ+nicSH2
p110δ+nicSH2+ pY

Fig. S7

Tab. S1 Summary of all peptides analyzed for p85 constructs, pY peptide binding, and lipid binding. A. All peptide exchange data for experiments with p85 constructs and pY peptide binding analyzed for the p110δ catalytic subunit are shown for four conditions at six time points. The six time points are labelled for the four different conditions tested. B. All peptide exchange data for pY peptide binding analyzed for the p85α regulatory subunit are shown for three conditions at six time points. The six time points are labelled for the four different conditions tested. C. All peptide exchange data for lipid binding experiments analyzed for the p110δ and p85α are shown for two conditions at three time points. The three time points are labelled for the two different conditions tested. The charge state (Z), maximal number of exchangeable amides (#D) residue start number (S), and residue end number (E) are displayed for every peptide. The relative level of exchange is colored according to the figure in the legend. The data listed is the average of two independent experiments.

Tab S1A. p110δ peptides

				Time	ΔABD-p110δ					p110δ+iSH2						p110δ+nicSH2						p110δ+nicSH2 + pY					
Z	#D	S	E	3_0C	3	10	100	1000	3000	3_0C	3	10	100	1000	3000	3_0C	3	10	100	1000	3000	3_0C	3	10	100	1000	3000
1	3	7	11							33%	70%	56%	54%	59%	54%	25%	52%	55%	53%	55%	53%	13%	46%	52%	52%	53%	54%
1	6	12	19							47%	66%	57%	55%	59%	57%	48%	69%	60%	57%	58%	55%	41%	64%	59%	57%	55%	56%
1	8	21	31							1%	2%	6%	13%	18%	24%	2%	3%	7%	13%	17%	23%	1%	3%	7%	14%	17%	23%
1	5	24	31							2%	4%	10%	17%	27%	36%	2%	4%	10%	16%	25%	35%	2%	3%	9%	16%	23%	32%
1	8	32	42							4%	13%	20%	29%	32%	30%	4%	13%	21%	28%	30%	29%	2%	15%	19%	28%	29%	29%
3	16	43	61							2%	4%	8%	16%	24%	27%	2%	4%	10%	17%	23%	28%	1%	4%	8%	16%	22%	26%
4	16	43	61							2%	5%	8%	15%	25%	27%	2%	4%	8%	16%	25%	27%	2%	4%	7%	14%	22%	26%
3	19	71	91							6%	15%	24%	37%	54%	62%	6%	13%	23%	35%	49%	59%	4%	12%	21%	35%	47%	57%
3	8	86	96							3%	9%	16%	24%	45%	50%	1%	7%	12%	20%	38%	44%	0%	4%	11%	19%	35%	45%
2	16	120	138	7%	18%	17%	22%	26%	26%	5%	13%	14%	21%	27%	25%	5%	13%	15%	21%	23%	24%	4%	14%	15%	21%	25%	25%
3	16	120	138	10%	19%	20%	24%	27%	28%	7%	13%	17%	23%	28%	27%	6%	12%	16%	23%	25%	25%	5%	12%	17%	22%	24%	26%
4	16	120	138	9%	17%	18%	23%	27%	27%	7%	12%	15%	22%	28%	25%	6%	11%	16%	22%	24%	24%	5%	11%	15%	22%	24%	25%
2	8	128	138	12%	23%	24%	35%	41%	39%	8%	16%	21%	32%	41%	37%	8%	16%	23%	34%	39%	38%	6%	16%	22%	33%	38%	38%
2	9	128	139	10%	20%	21%	28%	32%	30%	9%	17%	20%	27%	33%	30%	7%	15%	19%	26%	29%	29%	6%	13%	18%	26%	29%	29%
2	5	139	145	-2%	1%	0%	21%	45%	44%	0%	-1%	1%	17%	44%	42%	0%	-1%	0%	19%	48%	46%	-2%	0%	3%	16%	47%	47%
2	11	150	162	6%	13%	17%	23%	31%	33%	6%	15%	16%	22%	32%	32%	4%	10%	17%	22%	31%	33%	4%	11%	18%	21%	30%	34%
2	13	150	164	1%	6%	9%	18%	28%	30%	1%	5%	10%	16%	28%	30%	1%	4%	11%	16%	25%	29%	1%	5%	9%	16%	24%	28%
3	13	150	164	1%	6%	9%	19%	29%	32%	1%	5%	9%	17%	29%	31%	1%	4%	9%	15%	25%	29%	1%	3%	8%	15%	24%	29%
1	3	160	164	3%	4%	9%	6%	7%	9%	6%	5%	4%	8%	8%	9%	-2%	-3%	-1%	5%	-2%	2%	-1%	-2%	-3%	-1%	-3%	3%
2	11	163	177	14%	22%	25%	37%	44%	45%	14%	21%	25%	36%	45%	45%	12%	21%	25%	35%	42%	44%	11%	20%	24%	35%	41%	43%
2	16	163	182	25%	34%	34%	43%	48%	49%	23%	33%	35%	43%	50%	49%	23%	33%	35%	43%	47%	48%	18%	32%	34%	42%	46%	48%
3	16	163	182	26%	34%	35%	44%	48%	48%	25%	34%	35%	43%	50%	48%	24%	34%	36%	42%	46%	48%	20%	33%	35%	42%	46%	48%
3	14	165	182	29%	39%	39%	49%	54%	53%	27%	38%	39%	48%	56%	53%	27%	37%	40%	48%	52%	52%	21%	36%	39%	47%	52%	53%
1	7	183	191	35%	43%	41%	54%	66%	63%	32%	44%	41%	50%	65%	61%	35%	47%	47%	54%	65%	65%	31%	45%	44%	52%	62%	64%
2	7	183	191	36%	47%	43%	55%	66%	63%	33%	45%	42%	52%	67%	63%	33%	45%	44%	53%	66%	63%	28%	44%	43%	51%	61%	63%
1	7	192	200	15%	20%	22%	32%	38%	36%	17%	22%	25%	35%	42%	38%	18%	21%	26%	36%	41%	39%	18%	22%	26%	35%	42%	40%
2	7	192	200	17%	23%	25%	33%	39%	37%	17%	23%	25%	34%	42%	39%	17%	21%	25%	33%	40%	38%	16%	21%	23%	29%	35%	36%
2	10	192	203	17%	32%	35%	42%	46%	44%	17%	31%	37%	42%	48%	45%	16%	31%	37%	42%	47%	45%	10%	26%	35%	41%	45%	45%
2	11	203	216	3%	10%	14%	30%	39%	39%	3%	8%	13%	28%	39%	39%	3%	8%	14%	28%	37%	39%	2%	7%	13%	26%	34%	37%
1	8	206	216	0%	6%	13%	34%	46%	46%	2%	6%	13%	32%	46%	46%	1%	5%	14%	33%	45%	46%	2%	5%	12%	30%	40%	44%
2	8	206	216	1%	6%	14%	35%	48%	48%	1%	4%	11%	32%	47%	47%	1%	4%	12%	32%	45%	47%	0%	3%	9%	29%	40%	45%
4	14	221	238	22%	42%	46%	52%	59%	54%	21%	39%	46%	51%	54%	54%	19%	38%	46%	51%	56%	53%	12%	33%	44%	49%	54%	54%
2	7	228	238	24%	47%	51%	61%	66%	63%	23%	44%	53%	61%	70%	65%	23%	45%	54%	61%	66%	65%	15%	38%	51%	59%	65%	65%
2	10	239	250	2%	5%	3%	4%	8%	11%	2%	5%	3%	3%	9%	10%	1%	4%	3%	3%	7%	10%	1%	5%	4%	3%	6%	10%
3	10	239	250	2%	5%	3%	4%	9%	11%	2%	5%	3%	3%	9%	10%	2%	4%	3%	3%	8%	10%	2%	5%	3%	3%	7%	10%
1	6	251	259	2%	4%	3%	3%	4%	4%	2%	4%	3%	3%	4%	3%	2%	4%	2%	3%	3%	3%	2%	5%	5%	6%	8%	5%
2	6	251	259	2%	4%	2%	2%	3%	3%	2%	3%	2%	2%	3%	3%	1%	4%	2%	2%	2%	2%	1%	4%	2%	2%	2%	2%
1	3	260	264	1%	2%	2%	4%	13%	23%	1%	1%	2%	3%	14%	24%	1%	2%	1%	2%	13%	20%	1%	2%	2%	2%	10%	19%
2	7	265	274	7%	13%	11%	16%	22%	23%	6%	11%	11%	15%	23%	23%	5%	11%	11%	16%	20%	22%	3%	10%	12%	16%	19%	23%
3	14	267	283	0%	3%	3%	4%	8%	9%	0%	5%	5%	3%	8%	9%	0%	4%	3%	3%	6%	9%	0%	3%	4%	3%	5%	8%
4	14	267	283	2%	6%	4%	5%	9%	11%	2%	5%	4%	4%	10%	11%	3%	4%	5%	6%	11%	13%	3%	4%	5%	6%	11%	12%
1	7	275	283	1%	3%	3%	6%	14%	16%	1%	3%	3%	6%	14%	15%	1%	3%	3%	5%	12%	14%	1%	3%	3%	4%	10%	13%
2	7	275	283	1%	2%	2%	7%	15%	16%	0%	2%	2%	6%	14%	14%	0%	2%	2%	5%	12%	15%	0%	2%	2%	5%	11%	13%
2	8	275	284	1%	3%	3%	8%	22%	24%	0%	3%	2%	6%	20%	22%	0%	5%	5%	8%	20%	21%	1%	4%	5%	8%	17%	20%
1	5	317	324	3%	10%	20%	32%	34%	35%	3%	10%	22%	32%	35%	36%	3%	9%	22%	32%	33%	35%	2%	7%	19%	32%	33%	35%
2	5	317	324	4%	11%	23%	32%	33%	35%	4%	12%	24%	32%	34%	38%	4%	13%	23%	30%	32%	36%	3%	9%	21%	31%	31%	36%
1	7	317	326	2%	7%	14%	23%	24%	25%	2%	7%	16%	22%	25%	26%	2%	6%	16%	22%	24%	25%	1%	5%	14%	23%	23%	25%
2	7	317	326	2%	7%	14%	23%	24%	25%	2%	7%	16%	22%	24%	26%	2%	6%	16%	22%	23%	25%	1%	5%	14%	22%	23%	25%
2	9	327	337	4%	14%	20%	22%	32%	33%	1%	8%	12%	23%	33%	30%	1%	5%	10%	21%	30%	29%	0%	4%	9%	21%	29%	29%
1	8	328	337	3%	21%	21%	27%	40%	38%	0%	13%	13%	26%	40%	36%	0%	14%	14%	27%	37%	36%	1%	13%	12%	27%	37%	37%
2	8	328	337	4%	15%	22%	27%	42%	40%	2%	8%	15%	28%	42%	39%	2%	6%	13%	26%	38%	37%	2%	7%	12%	26%	37%	38%
1	4	342	347	1%	2%	1%	1%	1%	2%	1%	2%	0%	0%	1%	1%	1%	1%	-1%	0%	0%	0%	0%	1%	0%	0%	0%	0%
1	9	342	352	3%	4%	4%	6%	10%	9%	3%	6%	4%	6%	9%	8%	2%	5%	5%	7%	11%	10%	2%	4%	3%	7%	9%	10%
2	11	342	354	2%	4%	6%	8%	9%	9%	2%	5%	7%	8%	10%	9%	0%	2%	4%	7%	10%	11%	1%	3%	6%	7%	9%	10%
2	10	354	365	10%	19%	22%	31%	39%	39%	9%	17%	21%	28%	41%	39%	8%	14%	20%	29%	36%	37%	6%	13%	20%	30%	36%	39%
1	9	355	365	12%	18%	22%	33%	40%	42%	10%	15%	21%	30%	43%	41%	10%	16%	22%	31%	39%	41%	7%	15%	19%	30%	38%	41%
2	9	355	365	14%	22%	24%	35%	46%	46%	12%	19%	23%	34%	47%	47%	11%	19%	25%	35%	43%	46%	8%	16%	22%	33%	40%	44%
3	11	364	377	4%	11%	15%	25%	35%	39%	4%	12%	16%	23%	34%	38%	4%	13%	17%	24%	33%	38%	3%	10%	16%	25%	32%	38%
2	9	366	377	3%	11%	15%	22%	34%	40%	3%	10%	16%	20%	33%	39%	3%	10%	16%	20%	30%	38%	2%	12%	19%	23%	27%	33%
3	9	366	377	3%	11%	14%	21%	34%	41%	3%	10%	15%	19%	33%	38%	3%	10%	16%	19%	30%	38%	2%	8%	14%	19%	27%	35%
3	10	378	390	4%	17%	26%	38%	43%	39%	4%	14%	26%	37%	43%	39%	4%	10%	23%	37%	40%	38%	3%	11%	28%	38%	40%	40%
1	3	388	392	4%	7%	6%	5%	6%	6%	1%	1%	1%	0%	2%	1%	0%	1%	1%	1%	2%	1%	1%	1%	1%	1%	2%	2%
2	11	426	438	5%	11%	14%	26%	32%	32%	5%	10%	14%	25%	32%	32%	4%	10%	14%	22%	31%	32%	3%	9%	12%	23%	30%	31%
3	11	426	438	5%	11%	16%	25%	31%	31%	5%	11%	15%	24%	31%	31%	5%	11%	15%	23%	29%	29%	2%	9%	11%	22%	25%	28%
2	10	439	452	16%	27%	24%	29%	32%	30%	15%	26%	24%	28%	33%	30%	11%	25%	25%	27%	31%	30%	10%	24%	25%	28%	31%	30%
2	9	440	452	19%	32%	29%	34%	38%	35%	17%	31%	29%	33%	39%	35%	13%	29%	30%	32%	36%	35%	12%	28%	30%	32%	35%	35%
2	12	453	468	15%	29%	36%	57%	66%	64%	10%	23%	34%	53%	69%	66%	7%	22%	34%	52%	64%	65%	6%	20%	32%	52%	64%	67%
1	3	471	475	0%	-1%	-1%	0%	0%	0%	-1%	-1%	-1%	-1%	-1%	0%	0%	-1%	-1%	-1%	-1%	0%	0%	0%	-1%	-1%	-1%	0%
2	10	471	485	2%	8%	14%	27%	36%	38%	2%	7%	14%	25%	37%	37%	2%	7%	14%	25%	35%	37%	1%	5%	13%	25%	34%	37%
1	6	476	485	2%	10%	18%	35%	49%	51%	2%	9%	16%	35%	50%	53%	2%	9%	18%	34%	48%	52%	1%	8%	14%	32%	44%	49%
2	6	476	485	3%	11%	17%	36%	50%	53%	3%	9%	17%	33%	51%	52%	3%	9%	17%	34%	49%	53%	2%	7%	14%	33%	47%	51%
2	10	512	523	20%	29%	27%	29%	33%	33%	20%	28%	27%	29%	36%	32%	21%	30%	28%	28%	34%	34%	22%	27%	26%	27%	32%	34%
2	6	516	523	46%	59%	54%	52%	59%	54%	45%	56%	55%	54%	61%	56%	46%	58%	59%	55%	58%	56%	43%	54%	56%	52%	54%	54%
1	4	524	529	0%	4%	3%	31%	40%	35%	0%	4%	4%	29%	40%	36%	-1%	3%	3%	1%	17%	17%	-1%	6%	4%	29%	37%	38%
3	20	524	546	0%	1%	2%	6%	11%	12%	0%	1%	2%	5%	11%	12%	0%	1%	2%	4%	7%	9%	0%	1%	2%	5%	9%	12%
4	20	524	546	0%	1%	2%	6%	10%	12%	0%	1%	2%	5%	10%	11%	0%	1%	2%	4%	7%	9%	0%	1%	2%	5%	9%	11%
5	20	524	546	0%	1%	2%	6%	11%	10%	0%	1%	2%	5%	11%	9%	0%	1%	2%	3%	7%	8%	0%	1%	2%	5%	9%	9%
3	14	530	546	0%	1%	2%	4%	5%	5%	0%	1%	2%	3%	5%	5%	0%	1%	2%	3%	4%	5%	1%	2%	3%	3%	4%	5%
4	14	530	546	0%	0%	1%	3%	4%	4%	0%	1%	1%	3%	4%	4%	0%	1%	1%	3%	4%	4%	0%	1%	1%	3%	4%	4%
2	13	550	564	0%	1%	1%	4%	7%	8%	0%	1%	1%	3%	8%	7%	0%	1%	1%	4%	7%	8%	0%	1%	1%	3%	6%	7%
3	13	550	564	0%	0%	0%	2%	5%	7%	0%	0%	0%	1%	6%	7%	0%	1%	1%	3%	6%	7%	0%	0%	1%	2%	4%	5%
1	4	568	574	0%	6%	13%	31%	57%	59%	0%	4%	12%	30%	62%	63%	1%	5%	16%	36%	56%	63%	5%	9%	20%	32%	54%	63%
1	3	575	579	-1%	10%	17%	50%	79%	84%	-2%	8%	16%	44%	77%	83%	-2%	8%	15%	43%	72%	79%	-2%	6%	13%	41%	70%	81%
1	3	591	595	0%	2%	8%	35%	58%	57%	0%	2%	10%	22%	60%	52%	0%	1%	6%	19%	45%	52%	0%	1%	5%	20%	46%	53%
3	11	596	608	2%	7%	8%	16%	20%	21%	2%	5%	7%	15%	20%	21%	2%	5%	8%	15%	18%	21%	2%	6%	8%	14%	18%	22%
4	11	596	608	6%	10%	10%	19%	23%	24%	5%	9%	10%	14%	22%	21%	4%	6%	9%	14%	18%	21%	4%	6%	8%	13%	17%	20%
1	8	616	625	3%	12%	17%	28%	36%	39%	3%	12%	17%	27%	38%	41%	5%	13%	19%	27%	37%	41%	5%	14%	19%	28%	37%	42%
2	8	616	625	4%	12%	17%	28%	36%	39%	5%	13%	18%	27%	38%	42%	4%	13%	18%	26%	37%	42%	3%	12%	19%	26%	37%	42%
1	3	647	651	-1%	0%	0%	0%	1%	1%	-1%	0%	-1%	0%	2%	0%	0%	-1%	-1%	0%	0%	-1%	2%	0%	0%	1%	0%	-1%
2	3	647	651	-1%	-1%	-1%	-3%	-2%	-2%	1%	0%	-1%	-2%	-2%	-2%	-1%	0%	-2%	-3%	-2%	-2%	0%	-1%	-1%	-3%	-2%	-2%
2	6	648	655	2%	3%	2%	6%	8%	9%	0%	2%	4%	8%	7%	6%	0%	1%	2%	8%	8%	7%	2%	4%	4%	10%	9%	9%
3	6	648	655	-1%	2%	1%	1%	3%	3%	0%	3%	2%	1%	2%	1%	-2%	2%	0%	-1%	1%	0%	-1%	1%	1%	0%	0%	0%
1	3	656	661	20%	23%	22%	31%	58%	61%	19%	21%	21%	27%	51%	59%	18%	26%	21%	28%	48%	58%	19%	23%	21%	26%	45%	56%
1	5	662	668	0%	0%	0%	1%	2%	4%	0%	0%	0%	0%	1%	3%	0%	0%	0%	0%	1%	2%	1%	1%	1%	1%	2%	4%

Tab S1A cont. p110δ peptides

				ΔABD-p110δ						p110δ+iSH2						p110δ+nicSH2						p110δ+nicSH2 + pY					
Z	#D	S	E	3_0C	3	10	100	1000	3000	3_0C	3	10	100	1000	3000	3_0C	3	10	100	1000	3000	3_0C	3	10	100	1000	3000
2	5	662	668	0%	0%	1%	2%	3%	5%	0%	0%	1%	1%	2%	3%	1%	0%	1%	1%	2%	3%	1%	1%	1%	1%	2%	3%
3	13	683	697	2%	1%	2%	5%	10%	15%	0%	0%	1%	1%	7%	12%	-1%	-2%	-2%	-1%	2%	9%	-1%	0%	0%	-1%	4%	8%
1	4	684	689	-1%	3%	2%	0%	8%	9%	-1%	2%	0%	1%	4%	4%	-1%	0%	0%	-1%	2%	2%	0%	1%	1%	0%	2%	3%
1	3	693	697	1%	2%	5%	2%	8%	8%	0%	2%	5%	3%	5%	5%	2%	5%	3%	0%	3%	4%	2%	6%	4%	1%	4%	6%
3	13	698	713	16%	31%	35%	43%	55%	50%	11%	23%	29%	38%	56%	46%	9%	22%	30%	40%	50%	49%	7%	19%	29%	38%	46%	48%
2	15	698	715	13%	26%	26%	35%	48%	46%	10%	21%	23%	31%	45%	43%	9%	20%	23%	30%	39%	42%	8%	19%	23%	30%	37%	41%
3	15	698	715	13%	26%	26%	35%	47%	46%	10%	20%	23%	31%	46%	43%	9%	20%	23%	30%	39%	42%	8%	19%	22%	29%	37%	41%
5	15	698	715	13%	26%	26%	34%	47%	45%	9%	20%	22%	29%	46%	41%	8%	19%	23%	29%	39%	41%	7%	17%	22%	28%	36%	40%
1	4	720	725	31%	39%	38%	59%	73%	69%	32%	36%	39%	51%	72%	75%	32%	37%	40%	50%	67%	74%	29%	38%	39%	52%	66%	73%
1	6	720	727	21%	40%	43%	62%	71%	66%	19%	34%	42%	53%	70%	66%	23%	35%	40%	51%	64%	65%	22%	38%	40%	51%	62%	64%
2	6	720	727	15%	26%	27%	53%	63%	62%	15%	24%	23%	47%	66%	66%	15%	22%	23%	43%	58%	63%	13%	22%	22%	42%	58%	62%
2	11	726	740	1%	2%	3%	13%	20%	22%	1%	1%	3%	12%	20%	21%	0%	1%	2%	11%	19%	21%	0%	1%	2%	11%	19%	21%
2	9	728	740	1%	2%	2%	16%	25%	26%	1%	2%	3%	14%	24%	25%	0%	1%	2%	13%	23%	24%	0%	1%	2%	14%	23%	24%
2	10	728	741	0%	0%	3%	14%	22%	23%	0%	0%	2%	13%	22%	22%	0%	0%	2%	12%	21%	22%	0%	1%	2%	12%	21%	22%
1	3	741	745	0%	-3%	-1%	0%	18%	31%	-2%	-4%	-1%	0%	19%	32%	-1%	1%	1%	6%	14%	36%	2%	5%	3%	6%	14%	39%
1	4	746	751	-2%	-2%	-1%	16%	33%	39%	-2%	-1%	-1%	15%	35%	39%	-3%	-2%	-1%	14%	29%	38%	-2%	-2%	-1%	13%	27%	40%
2	8	752	762	10%	11%	9%	12%	17%	18%	10%	11%	10%	10%	15%	17%	10%	12%	10%	10%	13%	15%	9%	12%	10%	10%	12%	15%
3	8	752	762	11%	14%	12%	14%	20%	22%	11%	14%	12%	12%	19%	20%	10%	14%	13%	12%	16%	20%	10%	14%	13%	12%	15%	19%
1	4	761	766	17%	25%	31%	39%	52%	49%	16%	25%	31%	38%	55%	51%	13%	24%	30%	36%	48%	50%	9%	22%	28%	38%	46%	51%
2	7	776	784	1%	10%	10%	15%	21%	17%	0%	7%	9%	13%	21%	16%	0%	6%	9%	12%	17%	20%	-2%	5%	8%	12%	16%	16%
1	3	789	793	1%	1%	1%	2%	2%	3%	1%	1%	1%	1%	2%	2%	0%	0%	0%	0%	1%	1%	0%	1%	1%	1%	1%	1%
4	15	799	816	12%	21%	20%	26%	33%	33%	12%	20%	21%	25%	35%	33%	12%	20%	21%	25%	32%	34%	10%	19%	21%	25%	31%	33%
2	5	801	807	0%	3%	4%	18%	23%	24%	-2%	2%	4%	15%	22%	22%	2%	6%	6%	20%	25%	25%	1%	4%	5%	16%	24%	24%
2	15	808	826	2%	4%	4%	7%	14%	17%	1%	3%	3%	5%	11%	15%	1%	3%	3%	5%	10%	14%	2%	3%	4%	4%	10%	14%
3	15	808	826	2%	4%	4%	5%	13%	17%	1%	2%	2%	3%	10%	14%	1%	2%	2%	3%	8%	13%	0%	2%	2%	3%	7%	12%
1	8	827	836	6%	15%	14%	20%	31%	34%	4%	11%	14%	17%	30%	30%	4%	11%	13%	17%	23%	27%	4%	10%	14%	17%	23%	28%
2	8	827	836	3%	10%	10%	17%	26%	31%	3%	10%	11%	16%	25%	29%	4%	12%	14%	18%	23%	27%	4%	11%	13%	18%	23%	29%
1	5	830	836	-1%	2%	0%	7%	24%	38%	-1%	3%	0%	3%	18%	33%	-2%	1%	1%	1%	12%	27%	0%	1%	0%	1%	13%	29%
1	3	840	844	63%	68%	67%	66%	69%	63%	65%	70%	70%	66%	73%	64%	60%	68%	70%	65%	67%	63%	62%	68%	68%	64%	67%	66%
1	4	845	850	73%	82%	79%	80%	81%	73%	72%	74%	78%	81%	84%	75%	70%	80%	80%	78%	78%	74%	67%	84%	87%	81%	75%	73%
2	5	850	856	17%	42%	43%	56%	64%	59%	17%	42%	47%	49%	64%	61%	15%	35%	41%	46%	56%	58%	14%	39%	44%	48%	58%	60%
2	10	856	868	13%	21%	26%	36%	41%	41%	14%	20%	27%	36%	43%	42%	13%	19%	25%	35%	39%	40%	13%	18%	25%	35%	39%	41%
3	10	856	868	14%	21%	27%	36%	42%	42%	14%	19%	26%	35%	43%	42%	13%	18%	25%	34%	39%	40%	13%	17%	24%	34%	38%	40%
2	9	857	868	16%	25%	31%	42%	45%	43%	16%	21%	30%	39%	46%	43%	15%	20%	29%	39%	42%	42%	14%	19%	27%	38%	41%	42%
1	4	869	874	0%	4%	0%	0%	6%	12%	0%	4%	1%	0%	6%	10%	0%	3%	1%	0%	4%	8%	0%	5%	1%	-1%	3%	9%
1	5	875	881	1%	0%	2%	1%	1%	2%	1%	0%	0%	0%	1%	1%	1%	0%	0%	0%	0%	1%	0%	1%	1%	1%	1%	1%
1	3	882	886	1%	0%	0%	1%	1%	2%	0%	1%	1%	1%	1%	1%	0%	2%	0%	1%	1%	2%	1%	1%	1%	1%	1%	1%
3	13	886	900	-1%	1%	2%	7%	6%	6%	-2%	0%	1%	5%	8%	5%	-1%	0%	1%	4%	5%	5%	-1%	1%	2%	5%	6%	6%
1	5	901	907	-1%	3%	3%	16%	25%	23%	0%	7%	2%	16%	29%	23%	-1%	6%	2%	12%	22%	22%	-1%	5%	1%	13%	21%	23%
2	5	901	907	-1%	4%	3%	17%	25%	23%	-2%	5%	3%	13%	28%	22%	-2%	5%	1%	10%	22%	21%	-2%	4%	2%	9%	22%	23%
1	6	908	915	3%	9%	14%	21%	29%	29%	3%	8%	15%	20%	30%	29%	3%	7%	14%	19%	24%	26%	3%	8%	16%	21%	27%	31%
2	6	908	915	1%	7%	12%	20%	28%	28%	2%	6%	12%	19%	28%	28%	1%	5%	10%	18%	22%	24%	2%	6%	11%	18%	24%	27%
2	10	908	919	5%	17%	23%	32%	36%	35%	6%	14%	22%	31%	37%	34%	4%	11%	20%	30%	33%	33%	4%	11%	21%	31%	34%	34%
3	10	908	919	5%	17%	23%	32%	36%	35%	6%	14%	22%	31%	37%	35%	4%	11%	20%	30%	33%	33%	4%	12%	20%	31%	34%	35%
2	9	909	919	6%	20%	27%	37%	40%	37%	7%	16%	26%	35%	40%	37%	5%	13%	23%	35%	37%	36%	4%	14%	24%	35%	37%	37%
3	9	909	919	6%	19%	27%	36%	40%	37%	7%	18%	27%	35%	41%	37%	5%	13%	23%	34%	37%	36%	5%	14%	24%	35%	37%	37%
1	5	920	926	59%	73%	64%	63%	67%	58%	50%	68%	59%	56%	73%	55%	45%	78%	66%	61%	64%	57%	42%	78%	67%	69%	68%	64%
4	13	920	935	20%	34%	37%	50%	57%	55%	18%	31%	35%	46%	58%	54%	14%	26%	30%	34%	45%	51%	12%	26%	32%	42%	51%	53%
4	18	939	958	7%	19%	19%	25%	33%	29%	7%	16%	18%	22%	33%	28%	7%	15%	16%	20%	25%	25%	6%	16%	18%	22%	26%	28%
5	18	939	958	7%	21%	20%	25%	32%	29%	7%	19%	19%	23%	32%	29%	7%	17%	16%	20%	24%	24%	7%	18%	19%	22%	26%	28%
2	9	964	974	1%	5%	7%	16%	20%	19%	-1%	2%	7%	11%	18%	17%	0%	4%	7%	12%	18%	17%	0%	3%	7%	13%	18%	19%
2	9	978	989	3%	10%	17%	46%	51%	49%	5%	11%	20%	48%	54%	51%	4%	9%	19%	46%	51%	50%	2%	8%	18%	45%	50%	50%
2	5	982	989	8%	17%	31%	66%	73%	69%	9%	15%	29%	65%	72%	70%	7%	13%	30%	66%	73%	71%	6%	12%	29%	63%	72%	70%
1	6	990	997	11%	20%	24%	30%	43%	49%	10%	17%	22%	29%	44%	42%	9%	17%	24%	29%	44%	42%	10%	16%	21%	28%	43%	43%
1	4	992	997	3%	24%	46%	65%	65%	64%	6%	22%	43%	63%	68%	62%	7%	19%	48%	63%	65%	63%	6%	13%	35%	61%	63%	63%
1	8	1001	1010	14%	31%	30%	44%	53%	51%	14%	26%	31%	41%	54%	51%	13%	27%	31%	40%	52%	50%	12%	27%	32%	39%	48%	48%
2	17	1001	1019	8%	13%	13%	21%	33%	40%	7%	11%	13%	20%	33%	39%	6%	11%	13%	20%	28%	33%	5%	10%	13%	19%	27%	34%
4	17	1001	1019	7%	11%	13%	20%	31%	38%	7%	11%	13%	19%	31%	36%	7%	11%	13%	18%	27%	32%	6%	10%	12%	18%	26%	33%
3	20	1001	1022	5%	9%	10%	19%	33%	39%	5%	8%	10%	16%	32%	38%	5%	8%	10%	14%	22%	27%	6%	10%	10%	15%	27%	33%
2	8	1010	1019	2%	1%	2%	3%	14%	24%	1%	1%	1%	3%	12%	21%	0%	0%	0%	2%	7%	13%	0%	0%	0%	2%	8%	15%
3	8	1010	1019	0%	1%	1%	6%	14%	22%	1%	1%	1%	3%	12%	20%	2%	1%	2%	3%	9%	13%	2%	1%	2%	3%	9%	15%
1	3	1023	1027	54%	78%	75%	67%	61%	64%	48%	78%	70%	64%	66%	63%	35%	52%	56%	59%	68%	66%	36%	66%	72%	69%	73%	68%
3	9	1023	1033	34%	50%	46%	48%	49%	45%	31%	49%	46%	46%	50%	45%	11%	26%	40%	48%	49%	48%	23%	46%	50%	50%	51%	50%
4	9	1023	1033	36%	55%	50%	50%	52%	49%	33%	53%	50%	49%	53%	49%	11%	25%	42%	48%	50%	49%	21%	47%	52%	51%	51%	51%
2	8	1024	1033	42%	56%	51%	53%	55%	52%	36%	52%	49%	50%	53%	49%	13%	26%	43%	50%	52%	50%	26%	47%	50%	51%	51%	51%
3	8	1024	1033	37%	53%	48%	49%	51%	49%	33%	51%	49%	48%	54%	49%	8%	25%	41%	49%	51%	49%	21%	46%	50%	50%	50%	50%
1	4	1028	1033	42%	64%	59%	64%	65%	64%	33%	76%	62%	69%	71%	67%	6%	36%	60%	69%	69%	68%	22%	65%	66%	69%	70%	67%
2	9	1034	1044	43%	51%	48%	49%	52%	48%	42%	48%	49%	48%	53%	48%	36%	48%	51%	48%	51%	48%	38%	48%	49%	48%	50%	50%
3	9	1034	1044	43%	49%	51%	51%	51%	49%	42%	47%	51%	52%	53%	49%	37%	46%	51%	51%	51%	49%	38%	46%	50%	50%	50%	49%

Relative % deuteration: 0 | 10 | 20 | 30 | 40 | 50 | 60 | 70

Tab S1B. p85α peptides

				p110δ+iSH2						p110δ+nicSH2						p110δ+nicSH2+pY					
Charge	MaxD	Start	End	3_0C	3	10	100	1000	3000	3_0C	3	10	100	1000	3000	3_0C	3	10	100	1000	3000
1	4	327	332							18%	31%	62%	74%	78%	81%	18%	32%	60%	73%	83%	83%
1	7	333	341							12%	22%	27%	43%	49%	47%	7%	18%	19%	30%	44%	46%
2	7	333	341							12%	22%	27%	42%	48%	47%	5%	17%	19%	29%	43%	46%
1	6	334	341							16%	30%	35%	51%	59%	56%	10%	27%	27%	38%	52%	56%
2	6	334	341							15%	29%	34%	50%	58%	55%	7%	24%	25%	35%	51%	54%
1	6	342	349							2%	9%	23%	36%	57%	57%	1%	11%	17%	31%	49%	53%
2	6	342	349							-3%	9%	15%	31%	56%	62%	-4%	4%	6%	21%	48%	56%
3	12	342	355							6%	14%	16%	23%	37%	41%	2%	8%	13%	20%	32%	36%
2	8	346	355							7%	18%	19%	21%	32%	32%	2%	10%	17%	20%	27%	31%
2	14	356	371							15%	20%	20%	24%	24%	25%	11%	17%	16%	20%	23%	24%
2	15	356	372							12%	15%	16%	18%	19%	19%	9%	13%	13%	15%	18%	18%
2	7	372	380							5%	14%	20%	24%	44%	49%	5%	14%	19%	22%	35%	41%
4	17	381	400							7%	15%	18%	23%	28%	27%	3%	9%	14%	20%	26%	27%
2	10	402	413							5%	12%	21%	21%	22%	22%	0%	4%	8%	17%	21%	21%
3	10	402	413							6%	12%	21%	21%	24%	24%	1%	4%	8%	17%	23%	23%
1	8	404	413							5%	17%	35%	30%	30%	28%	1%	10%	13%	25%	28%	30%
2	8	404	413							8%	15%	29%	27%	29%	28%	0%	5%	10%	21%	28%	28%
3	8	404	413							9%	19%	29%	29%	31%	30%	2%	8%	14%	24%	29%	29%
1	7	405	413							10%	22%	35%	35%	38%	37%	2%	10%	15%	30%	36%	36%
2	7	405	413							9%	23%	37%	37%	39%	37%	2%	11%	16%	30%	37%	36%
1	4	414	420							34%	56%	72%	75%	75%	72%	2%	12%	25%	59%	73%	73%
2	4	414	420							33%	55%	71%	74%	74%	72%	1%	11%	23%	58%	72%	72%
3	11	421	434							14%	23%	28%	37%	41%	43%	12%	19%	26%	34%	38%	39%
3	12	421	435							18%	27%	32%	38%	43%	44%	16%	23%	28%	35%	40%	40%
2	13	421	436							20%	30%	29%	35%	21%	40%	17%	32%	28%	34%	20%	37%
3	13	421	436							16%	30%	29%	31%	30%	36%	19%	35%	28%	30%	30%	32%
4	20	421	443							22%	34%	39%	45%	48%	47%	21%	34%	38%	44%	46%	46%
1	6	436	443	43%	57%	58%	59%	66%	59%	27%	44%	54%	57%	59%	59%	31%	53%	53%	58%	59%	59%
2	11	444	456	1%	2%	5%	22%	47%	45%	1%	0%	1%	4%	16%	23%	1%	1%	2%	14%	32%	39%
3	11	444	456	2%	2%	5%	21%	47%	45%	1%	0%	2%	4%	16%	23%	2%	1%	3%	14%	32%	40%
2	8	467	476	1%	0%	1%	6%	27%	36%	1%	0%	1%	2%	15%	28%	0%	0%	1%	3%	16%	28%
3	9	477	487	1%	1%	1%	2%	9%	13%	1%	1%	0%	1%	5%	9%	1%	1%	0%	1%	4%	9%
2	5	487	493	-7%	-1%	-1%	-4%	-4%	-2%	-7%	-6%	5%	-5%	-4%	-4%	-8%	-1%	-1%	3%	-4%	-4%
2	7	487	495	0%	0%	0%	1%	3%	4%	0%	0%	0%	1%	1%	1%	0%	0%	0%	0%	0%	3%
2	6	488	495	2%	1%	0%	0%	2%	3%	4%	4%	-1%	-1%	0%	1%	4%	2%	0%	-1%	0%	0%
4	15	521	537	1%	4%	4%	10%	26%	25%	1%	3%	2%	9%	22%	24%	1%	1%	2%	7%	19%	23%
3	10	538	549	0%	-1%	2%	1%	12%	21%	0%	0%	0%	1%	9%	20%	0%	0%	-1%	0%	7%	15%
3	9	546	556	1%	1%	0%	2%	19%	19%	0%	1%	0%	0%	5%	9%	0%	1%	-1%	0%	5%	10%
3	12	556	570	0%	1%	1%	7%	23%	32%	1%	0%	1%	3%	10%	16%	0%	0%	1%	4%	11%	18%
4	11	557	570	0%	1%	1%	8%	24%	34%	0%	0%	0%	3%	10%	16%	0%	0%	1%	4%	12%	18%
2	9	571	581	0%	0%	1%	5%	29%	38%	0%	0%	0%	1%	10%	17%	0%	0%	0%	2%	13%	24%
3	9	571	581	1%	2%	2%	8%	30%	38%	2%	1%	1%	2%	11%	19%	1%	1%	1%	3%	15%	25%
3	6	574	581	0%	4%	1%	4%	32%	38%	0%	3%	1%	0%	8%	12%	0%	3%	1%	1%	12%	24%
1	3	597	601							28%	46%	46%	48%	51%	48%	30%	44%	49%	47%	50%	51%
1	8	597	606							28%	42%	39%	36%	40%	35%	23%	39%	36%	34%	36%	33%
2	8	597	606							35%	40%	44%	55%	50%	47%	30%	41%	44%	54%	55%	45%
3	25	610	637							4%	10%	13%	17%	22%	22%	2%	8%	10%	13%	15%	17%
4	25	610	637							4%	11%	13%	18%	23%	23%	2%	8%	10%	13%	15%	17%
5	25	610	637							4%	11%	13%	17%	22%	22%	2%	8%	10%	12%	15%	16%
3	21	614	637							4%	12%	14%	20%	26%	27%	2%	9%	11%	14%	17%	20%
4	21	614	637							4%	12%	15%	20%	26%	26%	1%	8%	11%	14%	17%	20%
1	8	647	656							23%	33%	36%	42%	48%	45%	9%	24%	27%	34%	45%	46%
2	8	647	656							28%	34%	40%	47%	49%	49%	10%	23%	29%	38%	47%	48%
1	9	647	657							20%	32%	34%	40%	49%	45%	9%	24%	24%	29%	39%	41%
2	9	647	657							18%	30%	29%	39%	46%	41%	8%	20%	21%	25%	37%	40%
3	9	647	657							21%	30%	31%	38%	46%	44%	12%	21%	21%	25%	39%	42%
1	3	657	661							-5%	-1%	-1%	-2%	-1%	2%	-6%	4%	-1%	-3%	-2%	0%
1	4	661	666							-1%	2%	1%	18%	26%	30%	-2%	0%	0%	13%	27%	29%
4	18	661	680							6%	12%	16%	22%	27%	27%	4%	9%	10%	18%	23%	25%
3	24	661	687							5%	10%	13%	20%	28%	30%	4%	9%	13%	22%	29%	31%
1	4	681	687							7%	15%	18%	30%	47%	52%	6%	19%	33%	52%	61%	60%
2	4	681	687							8%	20%	24%	32%	49%	54%	8%	23%	40%	55%	63%	63%
2	4	688	693							5%	26%	38%	38%	49%	57%	3%	17%	36%	38%	43%	50%
3	8	694	703							0%	2%	4%	5%	8%	9%	1%	3%	4%	5%	7%	7%
2	5	697	703							-2%	7%	7%	13%	17%	18%	-1%	9%	8%	11%	15%	15%
1	5	704	710							8%	16%	19%	29%	32%	30%	-1%	2%	1%	8%	16%	19%
2	5	704	710							9%	20%	22%	29%	34%	30%	0%	4%	3%	8%	18%	20%
1	4	711	716							-1%	-2%	-2%	2%	13%	16%	-1%	0%	-2%	3%	13%	16%
1	6	711	719							0%	0%	0%	2%	9%	12%	0%	1%	0%	2%	9%	10%
3	11	711	724							16%	22%	24%	28%	32%	33%	15%	21%	22%	27%	31%	32%
1	5	712	719							3%	6%	5%	7%	7%	9%	3%	5%	5%	7%	7%	7%
1	3	714	719							-1%	0%	1%	0%	5%	11%	-1%	2%	1%	1%	9%	1%

Relative % deuteration 0 10 20 30 40 50 60 70

Tab. S1C p110δ peptides

#D	Z	Start	End	p110δ+nicSH2 + pY 3	p110δ+nicSH2 + pY 30	p110δ+nicSH2 + pY 300	p110δ+nicSH2 + pY + liposomes 3	p110δ+nicSH2 + pY + liposomes 30	p110δ+nicSH2 + pY + liposomes 300
6	1	12	19	56%	56%	57%	56%	58%	61%
5	1	24	31	4%	17%	21%	2%	11%	21%
8	1	32	42	12%	25%	27%	9%	23%	27%
16	4	43	61	6%	14%	21%	5%	13%	19%
19	3	71	91	16%	36%	46%	20%	41%	58%
8	3	86	96	13%	14%	15%	11%	12%	17%
16	3	120	138	17%	25%	28%	19%	27%	30%
16	4	120	138	16%	28%	33%	18%	29%	35%
8	2	128	138	20%	34%	41%	21%	32%	41%
13	2	150	164	4%	14%	19%	4%	12%	18%
13	3	150	164	4%	15%	25%	3%	15%	26%
11	2	163	177	22%	35%	40%	24%	30%	39%
16	3	163	182	35%	46%	47%	34%	41%	47%
14	3	165	182	40%	51%	53%	39%	46%	52%
7	2	183	191	51%	56%	65%	49%	55%	65%
7	1	192	200	31%	42%	49%	29%	37%	49%
10	2	192	203	39%	50%	55%	35%	46%	52%
11	2	203	216	11%	27%	36%	8%	22%	31%
14	4	221	238	41%	58%	60%	34%	53%	58%
7	2	228	238	54%	71%	77%	47%	64%	74%
10	2	239	250	4%	4%	4%	3%	4%	4%
10	3	239	250	4%	5%	6%	4%	4%	5%
6	1	251	259	5%	3%	2%	3%	3%	3%
6	2	251	259	3%	3%	2%	2%	2%	2%
3	1	260	264	6%	8%	18%	4%	11%	18%
14	3	267	283	9%	10%	9%	6%	7%	8%
7	1	275	283	1%	4%	6%	1%	3%	6%
7	2	275	283	2%	5%	7%	2%	3%	6%
5	1	317	324	10%	35%	36%	7%	30%	36%
7	1	317	326	7%	25%	25%	4%	21%	25%
7	2	317	326	7%	26%	27%	6%	23%	27%
9	2	327	337	6%	24%	27%	5%	19%	24%
8	2	328	337	8%	25%	32%	7%	23%	29%
9	1	342	352	3%	5%	7%	2%	3%	6%
11	2	342	354	2%	8%	7%	3%	8%	9%
9	2	355	365	20%	33%	43%	18%	26%	37%
9	2	366	377	10%	24%	26%	8%	21%	30%
10	3	378	390	0%	1%	7%	6%	1%	8%
3	1	388	392	27%	28%	38%	28%	28%	37%
11	2	426	438	14%	20%	36%	14%	17%	33%
10	2	439	452	32%	36%	36%	29%	34%	37%
9	2	440	452	34%	40%	41%	30%	37%	41%
12	2	453	468	33%	52%	72%	35%	50%	70%
10	2	471	485	7%	22%	31%	6%	19%	30%
6	2	476	485	7%	23%	39%	6%	20%	36%
6	2	516	523	53%	53%	53%	51%	53%	53%
4	1	524	529	5%	15%	29%	2%	12%	32%
20	3	524	546	1%	6%	9%	2%	5%	8%
20	4	524	546	2%	9%	9%	2%	6%	10%
20	5	524	546	2%	6%	9%	2%	5%	9%
14	4	530	546	7%	7%	7%	1%	7%	6%
13	2	550	564	0%	1%	2%	0%	0%	1%
4	1	568	574	7%	25%	35%	5%	18%	35%
3	1	575	579	5%	28%	57%	5%	20%	56%
11	3	596	608	11%	18%	23%	10%	17%	23%
8	1	616	625	11%	26%	33%	8%	22%	32%
8	2	616	625	15%	27%	33%	16%	26%	33%
3	1	647	651	30%	29%	30%	35%	35%	38%
3	2	647	651	4%	4%	6%	5%	4%	9%
6	3	648	655	1%	-1%	3%	4%	4%	8%
5	1	662	668	4%	4%	2%	2%	1%	3%
5	2	662	668	1%	1%	1%	1%	1%	1%
4	1	684	689	3%	10%	4%	3%	15%	8%
3	1	693	697	8%	7%	7%	8%	7%	8%
15	3	698	715	18%	29%	33%	14%	23%	31%
15	5	698	715	18%	30%	33%	14%	24%	31%
4	1	720	725	33%	36%	52%	31%	33%	43%
6	1	720	727	19%	29%	50%	15%	23%	45%
11	2	726	740	3%	7%	18%	2%	6%	19%
3	1	741	745	-13%	-15%	-9%	-11%	-13%	-8%
4	1	746	751	0%	6%	21%	0%	3%	17%
8	2	752	762	13%	15%	14%	13%	13%	14%
4	1	761	766	22%	36%	35%	25%	29%	37%
7	2	776	784	17%	23%	29%	16%	23%	35%
3	1	789	793	3%	3%	3%	4%	2%	5%
15	4	799	816	21%	26%	29%	19%	25%	29%
5	2	801	807	5%	14%	28%	5%	12%	37%
15	2	808	826	4%	4%	6%	3%	4%	5%
15	3	808	826	3%	4%	6%	3%	4%	5%
8	1	827	836	7%	17%	17%	6%	14%	16%
8	2	827	836	11%	19%	23%	11%	18%	21%
5	1	830	836	2%	3%	7%	2%	1%	2%
4	1	845	850	91%	90%	93%	81%	86%	91%
5	2	850	856	24%	46%	49%	14%	21%	40%
10	2	856	868	25%	41%	41%	23%	34%	40%
10	3	856	868	24%	38%	42%	20%	33%	39%
4	1	869	874	10%	8%	13%	9%	9%	12%
5	1	875	881	1%	0%	1%	1%	0%	1%
3	1	882	886	3%	0%	2%	2%	1%	0%
13	3	886	900	3%	6%	7%	1%	3%	7%
5	2	901	907	7%	10%	24%	7%	9%	19%
6	1	908	915	10%	21%	19%	9%	15%	19%
6	2	908	915	8%	23%	19%	7%	11%	14%
10	2	908	919	14%	34%	34%	8%	16%	27%
10	3	908	919	14%	34%	35%	8%	15%	28%
13	4	920	935	25%	42%	49%	16%	26%	34%
18	5	939	958	14%	22%	23%	11%	15%	19%
9	2	978	989	8%	35%	53%	7%	23%	45%
5	2	982	989	13%	53%	79%	10%	37%	63%
17	4	1001	1019	11%	17%	22%	11%	14%	19%
8	3	1010	1019	3%	5%	7%	2%	3%	4%
9	4	1023	1033	64%	70%	69%	46%	54%	65%
8	3	1024	1033	63%	66%	64%	45%	50%	61%
9	2	1034	1044	53%	56%	53%	45%	53%	55%
9	3	1034	1044	49%	53%	50%	43%	49%	52%

p85α peptides

#D	Z	Start	End	p110δ+nicSH2 + pY 3	p110δ+nicSH2 + pY 30	p110δ+nicSH2 + pY 300	p110δ+nicSH2 + pY + liposomes 3	p110δ+nicSH2 + pY + liposomes 30	p110δ+nicSH2 + pY + liposomes 300
7	1	333	341	21%	26%	39%	20%	23%	33%
7	2	333	341	20%	27%	38%	18%	23%	33%
6	1	334	341	30%	37%	45%	29%	34%	40%
6	2	334	341	25%	31%	42%	22%	28%	36%
6	2	342	349	4%	17%	29%	4%	19%	25%
12	3	342	355	11%	21%	27%	8%	19%	27%
14	2	356	371	19%	23%	24%	17%	21%	24%
15	2	356	372	14%	18%	18%	13%	16%	19%
7	2	372	380	16%	23%	22%	14%	20%	24%
17	4	381	400	11%	21%	25%	9%	18%	22%
10	2	402	413	6%	18%	26%	5%	13%	20%
10	3	402	413	7%	19%	25%	6%	13%	22%
8	2	404	413	4%	20%	27%	3%	13%	22%
7	1	405	413	6%	21%	29%	5%	15%	22%
7	2	405	413	6%	21%	29%	5%	16%	23%
4	1	414	420	11%	43%	67%	7%	14%	42%
4	2	414	420	12%	44%	71%	9%	15%	44%
12	3	421	435	27%	41%	39%	28%	39%	43%
20	4	421	443	36%	47%	46%	34%	45%	45%
11	2	444	456	2%	12%	23%	1%	8%	27%
8	2	467	476	2%	4%	10%	2%	6%	20%
5	2	470	476	0%	7%	13%	0%	8%	30%
5	2	487	493	6%	7%	18%	5%	12%	12%
7	2	487	495	3%	2%	5%	3%	0%	7%
6	2	488	495	2%	1%	4%	2%	1%	4%
15	4	521	537	3%	7%	12%	2%	6%	11%
10	3	538	549	1%	1%	3%	1%	1%	6%
9	3	546	556	1%	2%	3%	1%	2%	5%
12	3	556	570	2%	6%	12%	2%	7%	18%
9	2	571	581	1%	3%	8%	1%	2%	11%
9	3	571	581	2%	3%	11%	2%	4%	14%
6	3	574	581	3%	3%	7%	4%	4%	11%
8	1	597	606	43%	50%	41%	44%	49%	47%
25	3	610	637	9%	16%	17%	8%	14%	18%
25	4	610	637	8%	15%	15%	7%	14%	15%
25	5	610	637	8%	16%	16%	7%	14%	16%
21	4	614	637	9%	9%	17%	7%	15%	18%
8	2	647	656	27%	36%	40%	26%	35%	41%
9	2	647	657	19%	26%	29%	19%	24%	29%
18	4	661	680	10%	19%	22%	10%	16%	23%
24	3	661	687	10%	21%	24%	10%	18%	25%
4	1	681	687	32%	54%	63%	29%	49%	66%
4	2	688	693	22%	40%	43%	19%	41%	46%
8	3	694	703	5%	9%	9%	4%	9%	11%
5	2	697	703	6%	12%	13%	6%	12%	14%
5	1	704	710	2%	5%	15%	1%	2%	10%
5	2	704	710	3%	5%	14%	2%	4%	11%
6	1	711	719	2%	3%	8%	2%	3%	6%
11	3	711	724	29%	36%	43%	30%	35%	43%

Relative % deuteration

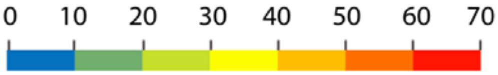

SI Materials and Methods

Protein expression and purification.

The ΔABD-p110δ was expressed as previously described (Berndt et al., 2010). In short for expression of all protein complexes 1 to 8 liters of *Spodoptera frugiperda* (Sf9) cells at a density of $1.0x10^6$ cells/mL were co-infected with an optimized ratio of viruses encoding complexes of the catalytic and regulatory subunit. The catalytic subunit contained an N-terminal 6xhis-tag followed by a TEV protease site, while the regulatory subunit was untagged. After 48-63 hr infection at 27˚C, cells were harvested and washed with ice-cold phosphate-buffered saline (PBS). The ΔABD-p110δ was expressed as a complex of the full length p110δ with a TEV protease site engineered after the ABD domain with the iSH2 domain of p85α The Sf9 cell pellets were lysed in 100 mls of 20 mM Tris pH-8.0, 100 mM NaCl, 5% glycerol, 10 mM Imidazole, and 2 mM β-mercaptoethanol, with one complete EDTA free protease inhibitor tablet added(Roche). Cells were lysed with a 4 minute probe sonication followed by centrifugation for 30 minutes at 140,000 g. The supernatant was then passed through a 0.45 µm Minisart filter unit (Sartorius Biotech). This filtrate was then passed over one 5 ml HisTrap FF column (GE healthcare). The column was washed with up to 30 mM imidazole and then eluted with a gradient of 0-100% buffer B (20 mM Tris pH-8.0, 100 mM NaCl, 5% glycerol, 300mM Imidazole, and 2 mM beta-mercaptoethanol). The pooled fractions were then added onto a 5 ml Heparin HP column (GE healthcare) and washed with buffer C (20 mM Tris pH 8, 100 mM NaCl, 2 mM DTT) and eluted with a 0-100% gradient of buffer D (20mM Tris pH 8, 2 mM DTT, 1 M NaCl). The ΔABD-p110δ construct was generated by an overnight incubation with a 1:10 w/w ratio with TEV protease in the presence of 10 mM β-mercaptoethanol. All other p110δ/p85α heterodimers still contained the N-terminal his-tag, however kinase assays on p110δ/p85α nicSH2 heterodimers with and without the 6xhis-tag removed by TEV cleavage showed similar results (Data not shown). Both the ΔABD-p110δ or p110δ/p85α heterodimers were then concentrated to 1.5 ml using a amicon 50k centrifugal filter (Millipore) and injected on a Superdex 1660 gel filtration column (GE healthcare) pre equilibrated with buffer E (20 mM Tris pH-7.2, 50 mM ammonium sulfate, 1% ethylene glycol, and 5 mM DTT). Fractions were collected and concentrated to 30 µM using a amicon 50k centrifugal filter (Millipore) and then aliquoted and frozen in liquid nitrogen.

Lipid vesicle preparation

Vesicles were prepared by adding the lipid components together in chloroform and evaporating organic solvent under a stream of dry argon. The lipid film was allowed to dry for thirty minutes under vacuum, and was then resuspended in a 20 mM Tris pH-7.5, 100 mM KCl, and 1 mM EGTA solution. The lipids were first bath sonicated for ten minutes, and then subjected to ten cycles of freeze-thaw between liquid nitrogen and a 42 °C water bath. The liposomes were finally extruded eleven times through a 100 nm filter using a mini extruder (Avanti Polar Lipids). Vesicles were frozen at -80 ºC and used within one week of preparation.

Protein lipid FRET assays

Vesicles were prepared exactly the same as above except with the inclusion of 10% dansyl PS (Avanti polar lipids). Two different types of lipid vesicles were prepared, one with 5% PIP2 (5% brain PIP2, 20% brain PS, 10% Dansyl PS, 35% brain PE, 15% brain PC, 10% cholesterol, and 5% sphingomyelin), and one with 0% PIP2 with the extra lipid composed of brain PS (25% brain PS, all other components the same). Protein solutions were allowed to preincubate with 10 μM pY peptide for 10 minutes before addition of lipid vesicles. Vesicles were 5 fold diluted with 20 mM tris pH-7.5, 100 mM NaCl, to give a concentration of to 200 μg/ml. 5 μl of the lipid mixture was mixed with 5 μl of a varying concentration of protein constructs, giving a final buffer of 100 μg/ml lipid, 20 mM tris pH-7.5, 90 mM NaCl, 10 mM KCl, 100 μM EGTA. The protein lipid mixture was allowed to equilibrate for 15 minutes. Reactions were measured using a PHERAStar HTS microplate reader (BMG Labtech) using a 280 nm excitation filter with 350 nm and 520 nm emission filters to measure Trp and Dansyl-PS FRET emissions, respectively. The FRET signal shown on the Y-axis is ($I-I_0$), where I is the intensity at 520 nm, where I_0 is the intensity for a solution with lipid only (without protein).

Akt phosphorylation assay

Phoenix cells (HEK-derived cells, Orbigen) grown in 35 mm-diameter wells were transfected with pMIG vectors (Kulathu et al., 2008) expressing myc-p110δ and FLAG-p85α wild-type or mutant using Genejuice (Novagen) as described by the manufacturer. Cells were grown for 24 h, starved for

15 h in serum-free media and lysed with ice-cold lysis buffer [20 mM Tris-HCl pH 7.4, 150 mM sodium chloride, 1 mM EDTA, 1 mM EGTA, 1% Nonidet P40 (BDH laboratories), 2.5 mM sodium pyrophosphate, 10 mM sodium fluoride, 0.25% sodium deoxycholate, 1 mM AEBSF (Melford), 10 mM sodium orthovanadate and 1X protease inhibitors mixture (Roche)]. Cell lysates were sonicated, clarified by centrifugation and the amount of protein quantified by Bradford assay. Equal amounts of lysates in SDS buffer were separated by 4%-12% Bis-Tris acrylamide gels and transferred to a PVDF membrane for immunoblotting. Mouse antibodies recognizing Akt and phospho-Akt (Ser473) (Cell Signaling Technology), myc (Santa Cruz), FLAG (Sigma) and actin (Boster Biological Technology) were used as primary antibodies. Horseradish peroxidase (HRP)-conjugated anti-mouse antibody (Santa Cruz) was employed as a secondary antibody and the proteins detected with SuperSignal West Pico Chemiluminescent Substrate (ThermoScientific). Quantification of western blots was performed using GeneTools software (Syngene). Intervening unrelated samples between vector and p110δ/p85α constructs have been removed as indicated by dotted line in Fig 7.

Measurement of deuterium incorporation

Samples were thawed rapidly on ice and then injected onto an online HPLC system that was immersed in ice. The protein was run over an immobilized pepsin column (Applied Biosystems, Poroszyme®, 2-3131-00) at 100 µl/min, and collected over a C18 peptide trap (Michrom, TR1/25109/2) for five minutes. The trap was then switched in line with a 5 µm particle, 50 mm x 1 mm C18 column (Discovery sciences, 218MS5105) with a guard column and peptides were eluted by a 5-45% gradient of buffer A (0.1% formic acid) and buffer B (80% acetonitrile, 20% H_2O, 0.02% formic acid) over thirty minutes and injected onto a Sciex QStar Pulsar hybrid QqTOF (Applied Biosystems) which collected mass spectra from a range of 350 to 1500 m/z.

Lipid binding, nucleotide binding, and p85 point mutation experiments were performed with an online UPLC system which replaced the C18 peptide trap with a 1.7 µm particle van-guard pre column (Waters), and used an Acquity® 1.7 µm particle, 100 mm x 1 mm C18 column (Waters). The UPLC system allowed separations to be carried out with a 20 minute gradient from 5-45% B. Mass analysis of the peptide centroids was performed as described previously using the software DXMS (Sierra Analytics) and is described below (Fig S7) (Burke et al., 2008). For lipid binding experiments there

was concern that the lipid might cause problems with UPLC performance and possible ion suppression. Our experiments showed that the lipid was retained on the column throughout the run with no negative effects on UPLC column performance (Fig. S7). The entire UPLC setup was flushed with 80% acetonitrile for 30 min at the end of the experiment to fully elute all lipid. The UPLC setup allowed for a faster and more efficient peptide separation, however this did cause changes in back exchange rates compared to HPLC runs. For this reason none of the comparisions in the manuscript were performed between HPLC and UPLC experiments, and were only performed under similar experimental protocols.

Protein digestion and peptide identification

Different digestion conditions were employed to optimize the peptide digestion map. These optimizations included changing denaturant concentration, flow rate over pepsin, and denaturation time (See below for optimal conditions). Peptide identification was performed by running tandem MS/MS experiments using a Sciex QStar Pulsar hybrid QqTOF (Applied Biosystems). IDA (information dependent acquisition) MS/MS experiments were performed with Analyst 2.0 with a 1 second TOF MS scan from 350-1500 m/z followed by two 1.5 second product ion scans with a capillary voltage of 5300 V. Data was analyzed using Mascot software v. 2.2 (Matrix Science) to identify peptides based on fragmentation and peptide mass. The MS tolerance was set at 30 ppm with a MS/MS tolerance of 0.2 Da. All peptides with both a mascot score >10 and a parent mass error smaller than 20 ppm were analyzed by the DXMS software (Sierra Analytics). Any peptides with a mascot score from 10 to 20 were further manually checked to see if there were any possible false identifications, and any ambiguous peptides were deleted from the peptide pool. The total list of peptides were then manually validated by searching a non deuterated TOF MS scan to test for correct m/z state, and check for the presence of overlapping peptides. Although the DXMS software was used to automate the initial analysis aftwewards every peptide shown in the paper was manually verified at every timepoint and replicate to check for correct charge state, m/z range, presence of overlapping peptides, and proper retention time.

Mass analysis of peptide centroids

All selected peptides passed the quality control threshold of the DXMS software, and were then manually examined for accurate identification and deuterium incorporation. There was a correction for on-exchange made by adding deuterium after the quench buffer was added. This value was subtracted from all deuterium exchange experiments. For this reason some peptides have a negative value of exchange in Table S1. Attempts were made at generating a fully deuterated sample, but were not successful, and for this reason results are shown as relative levels of deuteration with no correction for back exchange as described previously (Iacob et al., 2009). The only correction made was correcting for the level of deuterium in the exchange buffer (82%) (66% in lipid binding experiments). The real level of deuteration will be ~25-35% higher than what is shown, based on tests performed with fully deuterated standard peptides. All experiments were repeated in duplicate, and we found that the average error was ≤0.2 Da for non-corrected data, and for this reason we consider all non corrected changes between conditions of greater than 0.5 Da significant. The resulting deuterium incorporation was graphed versus the on-exchange time. The 3 seconds at 0 °C time point was graphed as 1 second in all on-exchange graphs to fit all data to the same graph. Full deuterium exchange methodology is described (Figure S7), and all deuterium exchange data for all peptides analyzed for phosphopeptide / p85 constructs (~240 peptides) is shown in tabular form (Tab. S1A, S1B). For lipid binding experiments we used less protein to maximize the ratio of lipid to protein. For this reason fewer peptides were able to be followed, but still over 150 peptides between the catalytic and regulatory subunit were quantified for deuterium incorporation (Tab. S1C).

Supplemental references

Bondeva, T., Pirola, L., Bulgarelli-Leva, G., Rubio, I., Wetzker, R., and Wymann, M.P. (1998). Bifurcation of lipid and protein kinase signals of PI3Kgamma to the protein kinases PKB and MAPK. Science *282*, 293-296.

Fu, Z., Aronoff-Spencer, E., Wu, H., Gerfen, G.J., and Backer, J.M. (2004). The iSH2 domain of PI 3-kinase is a rigid tether for p110 and not a conformational switch. Arch Biochem Biophys *432*, 244-251.

Iacob, R.E., Pene-Dumitrescu, T., Zhang, J., Gray, N.S., Smithgall, T.E., and Engen, J.R. (2009). Conformational disturbance in Abl kinase upon mutation and deregulation. Proc Natl Acad Sci U S A *106*, 1386-1391.

Kulathu, Y., Hobeika, E., Turchinovich, G., and Reth, M. (2008). The kinase Syk as an adaptor controlling sustained calcium signalling and B-cell development. EMBO J *27*, 1333-1344.
